# Phylogenetic Co-Occurrence of ExoR, ExoS, and ChvI, Components of the RSI Bacterial Invasion Switch, Suggests a Key Adaptive Mechanism Regulating the Transition between Free-Living and Host-Invading Phases in Rhizobiales

**DOI:** 10.1371/journal.pone.0135655

**Published:** 2015-08-26

**Authors:** Mary Ellen Heavner, Wei-Gang Qiu, Hai-Ping Cheng

**Affiliations:** 1 Biochemistry Program, The Graduate Center, City University of New York, New York, New York, United States of America; 2 Biological Sciences Department, Hunter College, City University of New York, New York, New York, United States of America; 3 Biological Sciences Department, Lehman College, City University of New York, Bronx, New York, United States of America; Centre National de la Recherche, FRANCE

## Abstract

Both bacterial symbionts and pathogens rely on their host-sensing mechanisms to activate the biosynthetic pathways necessary for their invasion into host cells. The Gram-negative bacterium *Sinorhizobium meliloti* relies on its RSI (ExoR-ExoS-ChvI) Invasion Switch to turn on the production of succinoglycan, an exopolysaccharide required for its host invasion. Recent whole-genome sequencing efforts have uncovered putative components of RSI-like invasion switches in many other symbiotic and pathogenic bacteria. To explore the possibility of the existence of a common invasion switch, we have conducted a phylogenomic survey of orthologous ExoR, ExoS, and ChvI tripartite sets in more than ninety proteobacterial genomes. Our analyses suggest that functional orthologs of the RSI invasion switch co-exist in Rhizobiales, an order characterized by numerous invasive species, but not in the order’s close relatives. Phylogenomic analyses and reconstruction of orthologous sets of the three proteins in Alphaproteobacteria confirm Rhizobiales-specific gene synteny and congruent RSI evolutionary histories. Evolutionary analyses further revealed site-specific substitutions correlated specifically to either animal-bacteria or plant-bacteria associations. Lineage restricted conservation of any one specialized gene is in itself an indication of species adaptation. However, the orthologous phylogenetic co-occurrence of all interacting partners within this single signaling pathway strongly suggests that the development of the RSI switch was a key adaptive mechanism. The RSI invasion switch, originally found in *S*. *meliloti*, is a characteristic of the Rhizobiales, and potentially a conserved crucial activation step that may be targeted to control host invasion by pathogenic bacterial species.

## Introduction

The Gram-negative soil bacterium *Sinorhizobium meliloti* Rm1021 fixes nitrogen inside the root nodules produced by its plant host alfalfa, *Medicago sativa* [1,2,3,4], and is one of the best characterized symbiotic models of bacterium-plant interactions [1]. *S*. *meliloti* shares extensive genomic congruence with animal pathogens such as *Brucella suis*; more than 90% of their genes show at least 98% identity [5]. No known work to date has directly compared the full genomes of *S*. *meliloti* and the prototypical *Brucella* type, *B*. *abortus*. However, the high genomic similarity of *B*. *suis* and *B*. *abortus* has been conclusively demonstrated [6,7], implying a high similarity between *S*. *meliloti* and *B*. *abortus*. In addition, *S*. *meliloti* also shares a high degree of synteny with plant pathogens such as *Agrobacterium tumefaciens* [8]. These similarities suggest that our understanding of *S*. *meliloti* might also be a prime source of information about pathogenic bacterial invasion of both mammalian and plant hosts.

Effective *S*. *meliloti* invasion of alfalfa depends on a series of signal exchanges and reciprocal structural developments [1,2,9,10], and is required for the initiation of *S*. *meliloti*-alfalfa symbiosis [11]. At the site of the invasion, the infection chamber inside curled alfalfa root hairs, *S*. *meliloti* cells must produce a bacterial exopolysaccharide, succinoglycan, in order to invade the plant root hair cells [11]. The production of succinoglycan is inversely related to the production of flagella by the same *S*. *meliloti* cells. During this process, *S*. *meliloti* cells switch from flagella producing free-living cells to succinoglycan producing host-invading cells [12]. Following *S*. *meliloti* invasion into root hairs, continuous structural and metabolic modifications occur within both the bacterial and the plant cells, resulting in the formation of root nodules filled with *S*. *meliloti* bacteroids, which convert atmospheric dinitrogen to ammonia for alfalfa’s use as a nitrogen source [2,9].

The critical switch of *S*. *meliloti* from free-living to host-invading cells is linked to up or down regulation of the expression of hundreds of genes, which are represented by succinoglycan and flagellum genes [13,14]. This switch is controlled by the ExoR, ExoS, and ChvI signaling pathway [15,16], which we refer to herein for the first time as the “RSI invasion switch.” ExoR is expressed as a cytoplasmic precursor (ExoRp), the structure of which has been resolved through computational modeling [17]. ExoRp is secreted to the periplasm as a mature and functional protein (ExoR_m_) that interacts with the periplasmic domain of ExoS (ExoS_p_), a sensor kinase [16,18]. It has been experimentally demonstrated that ExoR_m_ can be digested by a yet unidentified periplasmic protease to yield a nonfunctional product ExoR_c20_ [16]. Our current model suggests that the proteolytic processing of ExoR_m_, which could be a target of environmental changes or host signals, relieves ExoR inhibition of ExoS, and thus activates the RSI switch [16].

ExoS and ChvI comprise a typical two-component regulatory system (TCS) that belongs to the EnvZ/OmpR family [11,19,20,21]. ExoS is a 595-residue protein with two transmembrane domains. The first transmembrane domain is in close proximity to its N-terminus (residues 48 to 67). A periplasmic domain follows between residues 68 and 278, where interaction with ExoR_m_ occurs, and is followed by the second transmembrane domain and a cytoplasmic histidine kinase [11,13]. ExoS activation, triggered by the loss of ExoR_m_ inhibition, leads to ExoS auto-phosphorylation in its highly conserved cytoplasmic kinase domain, downstream phosphorylation of the 240 residue transcriptional factor ChvI [11], and the activation and/or suppression of multiple lifestyle-associated genes [12,13,14,15,18,22,23,24]. This control mechanism not only prepares cells by switching from flagellum to succinoglycan production, but also regulates metabolism and cell envelope changes necessary for cellular differentiation into endosymbiotic nitrogen fixing bacteroids [14,20,25].

Individual orthologous components of the RSI invasion switch have been found in other bacteria, including mammalian and plant pathogens. The ExoR ortholog from the plant symbiont *Rhizobium leguminosarum* regulates exopolysaccharide production [26], which is similar to the role of *S*. *meliloti* ExoR [12,27,28,29]. The ExoR ortholog from the plant pathogen *Agrobacterium tumefaciens* regulates the production of succinoglycan and biofilm [30], and the acid induced type VI secretion system [31,32], both of which are essential for host invasion. Mutant screens have identified ExoS and ChvI orthologs in *Agrobacterium tumefaciens*, *Rhizobium leguminosarum*, *Bartonella henselae*, and *Brucella abortus* [33,34,35,36,37,38]. The *A*. *tumefaciens* ChvG(ExoS)/ChvI system regulates virulence against its plant hosts by modulating succinoglycan and other virulence factor production based on the low pH at the site of infection [31,33,39,40]. The *R*. *leguminosarum* ChvG(ExoS) sensor kinase contributes to the control of outer membrane protein expression [35,41]. In *B*. *henselae*, the orthologous BatR/BatS TCS system responds to host-dependent environmental conditions and induces a virulence regulon necessary for intra-erythrocytic mammalian infection [34]. The BvrS(ExoS)/BvrR(ChvI) system in *B*. *abortus* regulates its invasion of host macrophages [36,37,42,43,44,45]. More recently, whole genome sequencing has identified a large number of putative ExoR, ExoS, and ChvI homologs in numerous other bacteria, many of which lack functional analysis. Most importantly, the discovery of individual components of the pathway in various mammalian and plant pathogens suggests that ExoR, ExoS, and ChvI may regulate host invasions that have otherwise been considered unique, and invasions that eventually bring harm rather than benefit to hosts.

In this report, we characterize the phylogenetic distribution of orthologs of the RSI pathway proteins to test if the presence of an “RSI Switch” is associated with an intracellular bacterial lifestyle. We take the approach of phylogenetic profiling, a bioinformatics method for identifying protein networks based on phylogenetic co-occurrence of network components [46,47,48]. Our findings demonstrate that functionally orthologous RSI invasion switches are only a pervasive, taxon-specific genomic characteristic among Rhizobiales. The ecological characteristics of the bacteria within this “RSI group” suggest that this signaling pathway is a key adaptive mechanism responsible for the success of both symbiotic and pathogenic dimorphic species. Furthermore, we have identified variable regions within ExoR and ExoS that are the best candidates for site-specific mutational analysis to further investigate the function of the RSI switch and its orthologous pathways in the Rhizobiales.

## Materials and Methods

### Phylogenomic analysis with BLAST

Best-best searches in the Kyoto Encyclopedia of Genes and Genomes (KEGG) Sequence Similarity Database (SSDB) [49,50] were conducted to define the phylogenomic limits of our queries. Sequences identified in phmmer Hidden Markov model (HMM) searches (Janelia Farms [51], GenBank nr database [52], E <= 1e^-5^ criterion, Alphaproteobacteria) were selected for further analysis. Context Specific (CS) protein blasts [53,54] were used to confirm the phmmer results. CS searches were implemented in three rounds with E = 10^–6^, 10^–5^, and 10^–4^. Reciprocal best NCBI blastp [55] searches were conducted against the GenBank [52] nr database (BLOSUM62, alignments of >= 60% of query length and E <= 1e^-5^). GenBank accession numbers and Blast results are provided in S1 Table. Many sequences demonstrated similarities to ExoS primarily within its highly conserved BaeS histidine kinase domain [CDD: COG0642]. Since the physical interaction of ExoR and ExoS within Rm1021 periplasmic space is crucial to the function of the RSI switch, we required significant similarities and alignments against ExoS periplasmic sensing domain (60% of ExoS length, E <= 1e^-5^). Genomic loci architectures and their associated COGs (Cluster of Orthologous Groups) were obtained from the Integrated Microbial Genomes and Metagenomes (IMG) database (http://img.jgi.doe.gov/) [56]. COG assignments were based primarily on those of Rm1021 and on consensus Rhizobiales COGs when differences were found.

To confirm that our criterion for putative homologs (i.e., E <= 1e^-5^) was sufficiently generous, we demonstrated that the predicted folds of excluded hits differed from the fold of ExoR. To guard against algorithm-intrinsic biases in this analysis, we utilized consensus predictions from four fold-threading servers (SPARKS-X [57], LOOPP [58,59,60,61,62], HHpred [63], and pGenThreader [64]).

When selecting species/strains from multiple sequences within a family or taxon for further investigation, we prioritized candidates based on following hierarchy: (1) type strains, (2) community selected reference organisms, and (3) the organism with the most publically available data.

### Filtering by structural characterization

Structural protein predictions were used to narrow our search for orthologs (results presented in S2 Table). For the identification of ExoR orthologs we required positive predictions of (1) Sel1 domains and (2) extracellular localization. Both of these sequence characteristics would be necessary for any ortholog to function in a manner similar to to ExoR. For ExoS orthologs, we required predictions of structural elements based on the *S*. *meliloti* prototype: a Sensor Domain [Pfam: PF13756] sandwiched between two trans-membrane helices (TMHs). ChvI putative orthologs were selected using the same methodology, requiring consensus predictions of (1) Response_reg [Pfam: Pf00072] and (2) Trans_reg_C [Pfam: PF00486] domains.

For structural predictions, we utilized a majority rule method to avoid intrinsic algorithm bias. Predictions for highly helical proteins such as ExoR can return inconsistent results as multiple sequence motifs that can fold into similar local structures (e.g., Sel1 and TPR domains). Accordingly, we used the consensus from (1) SMART [65,66], (2) Pfam [67], (3) phmmer [51], and (4) NCBI’s Conserved Domain Database (CDD) [68] to predict domains. Consensus trans-membrane predictions were compiled from (1) SMART [65,66], (2) HMMTOP [69], (3) Octopus [70], (4) Phobius [71], and (5) TMHMMv2.0 (http://www.cbs.dtu.dk/services/TMHMM/). Consensus secretion signals were obtained using (1) SMART [65,66], (2) Octopus [70], (3) SignalP4.0 [72], and (4) Phobius [71]. Protein localization and predictions of non-canonical secretion were based on PSORTb3.0.2 [73], the SOSUI prokaryota database [74], and the SecretomeP 2.0 algorithm [75].

### Multiple sequence alignments (MSAs)

MSAs were created using TCoffee Espresso with standard parameters [76]. MSA quality was verified by visual inspection using Jalview [77] and GenDoc [78] editors. The ExoR ortholog MSA was modified on the N-terminus to align the first Sel1 repeats and signal peptides. The ExoS MSA was trimmed into two regions, periplasmic and cytoplasmic, after the domains and 2D structure alignments were confirmed. No modifications were made to the ChvI alignment after manual verification.

The ExoR, ExoS, and ChvI alignments were blocked, removing all gaps greater than ten residues and increasing the percent continuous conserved sequence (TrimAl v1.3 [79] with ‘GappyOut’ parameters). Additional manual blocking of the ExoR ortholog alignment was made to (1) remove inter-domain non-conserved sites and (2) to assure the inclusion of predicted protein-protein interaction sites. 0.7% undetermined and gapped positions was achieved for ExoR. No modifications were made to the ExoS or ChvI TrimAl results. The final blocked alignments for ExoS and ChvI had 0.02% and 0.2% undetermined and gapped sites, respectively.

### Phylogenetic analyses

The ExoR, ExoS, and ChvI ortholog trees were reconstructed with maximal likelihood (RAxML) and Bayesian (MrBayes) algorithms. Since the histidine kinase domain of ExoS is highly conserved, we utilized only the periplasmic region for our phylogenetic analyses [80,81]. ProtTest2.4 [82] was used to rank models based on the blocked alignments using site-built BioNJ trees and AICc criteria. LG+G models were used for all of the data sets and invariant sites were not used as per XSEDE recommendations for RAxML-HPC. WAG+I+G, WAG+G, and JTT+G, were used for ExoR, ExoS, and ChvI ortholog sets, respectively, in MrBayes 3.2.1. Maximum likelihood trees (RAxML-HPC on XSEDE, 7.6.3) [83,84] were built with 1200 initial bootstraps and then to convergence on the CIPRES Science Gateway V.3.3 [85] (http://www.phylo.org/index.php/portal/#). Final bootstrapping was performed to auto majority rule (autoMRE) criterion and SumTrees Python script from the DendroPy3.12.0 package [86] was used to build the consensus trees. Bayesian trees [87,88,89] were built for all sequence sets on the City University of New York High Performance Computer Center (CUNY HPCC) cluster. Four rate categories for all analyses and invariant sites for ExoR orthologs were used. Final Bayesian phylogenies were run for 5 million generations with burn-ins of 750,000, producing consistent convergence for all ortholog sets.

For parsimony inference of gene gains and losses, we used a customized Perl script based on BioPerl [90] to extract the phylogeny of the selected Proteobacteria genomes from a bacterial species tree inferred using 16S RNA sequences [91]. FigTree (http://tree.bio.ed.ac.uk/software/figtree/) was used to produce tree images. We have presented pseudo-rooted protein phylogenies as this was more amenable for branching comparisons. Since the RSI protein branching patterns follow accepted Rhizobiales phylogenies [92,93], we have rooted according to the earliest predicted speciation within our dataset. We estimated levels of sequence conservation at each amino-acid position using the computer program Rate4Site [94]. This program outputs standardized scores of amino-acid substitution rates at each residue position based on a multiple protein sequence alignment. The lower the rate score, the more conserved a residue position is evolutionarily. We used *S*. *meliloti* orthologs as the reference molecule in this analysis.

## Results

### RSI-like pathways are unique to Alphaproteobacteria

In an initial survey, 394 and 398 bacterial genomes that putatively encode Rm1021 ExoS and ChvI orthologs (KEGG best-best analysis, orthology groups K14980 and K14981), respectively, were identified. The lack of KEGG one-to-one ExoS to ChvI putative ortholog matches, as would be expected, is likely due to incomplete annotations and/or the presence of orphan sensor kinases [95]. 39% and 51% of the ExoS and ChvI best-best hits, respectively, were from Alphaproteobacteria. Overall, slightly less than half (41%) of the KEGG hits were from the Rhizobiales, an order of Alphaproteobacteria. In contrast, Rm1021 ExoR initial ortholog searches (KEGG best-best orthology group K07126) returned only 83 genomes within 23 bacterial genera (100% Alphaproteobacteria and 92% Rhizobiales). These results strongly suggested that (1) genomes that encode ExoR orthologs are a subset of ExoS(ChvG)/ChvI genomes and (2) complete, orthologous RSI pathways could only be found in the Alphaproteobacteria. It is likely that outside of the Rhizobiales, TCSs similar to ExoS/ChvI function either without a third partner or with proteins other than ExoR orthologs.

Our curated ortholog searches first focused on Rm1021 ExoR, since this protein, in comparison to ExoS and ChvI, demonstrated the most limited similarity across Alphaproteobacteria genomes. The initial candidate orthologs (non-paralogous sequences from 95 genomes) were taken from HMM/CS BLAST results (Table 1 and S2 Table). However, this group was narrowed from 95 to the 92 genomes that were found to encode candidate orthologs of all three RSI proteins. Manual best-best BLASTs querying the putative ExoR orthologs against the *S*. *meliloti* Rm1021 genome, were positive for 57 of the 92 genomes (62%) (Table 1). Among this set of 57 candidate ExoR orthologs, 86% originate from Rhizobiales genomes, while the remaining 14% predominantly originate in the Rhodobacterales, a closely related, but potentially non-monophyletic order [93] (Table 1 and S2 Table). We concluded that although systems with proteins like ExoS and ChvI are widespread in Alphaproteobacteria, complete orthologous RSI-like pathways are only consistently found in a subset of this bacterial class.

**Table 1:** The identification of ExoR orthologs.

Selection methods	Criteria	Number of candidates
HMM & CS BLASTs	Query with Rm1021 ExoR	95
HMM & CS BLASTs	Genomes also encode candidate ExoS & ChvI orthologs	92
Reciprocal BLASTp	Manual best-best hits: ExoR to candidate sequences	57
Consensus structural predictions	Putative ExoR orthologs (1) with signal peptides, (2) without TMHs, & (3) without non-Sel1 domains	52
Phylogenetics: ML & Bayesian	Resolved with majority support	47, final set

BLAST queries were used to select putative homologs and the genomes with BLAST hits for all three RSI switch proteins were subsequently identified. Using reciprocal best-best searches, we then differentiated putative orthologs from homologs. The final set of RSI orthologs was obtained by (1) imposing strong predictions for functionality using localization signatures and domain architectures, and (2) requiring resolved ortholog phylogenies. (ExoS ortholog alignment blocking for reconstruction resulted in one set of redundant sequences among the Brucellaceae and reduced the non-redundant number of sequences to 46.)

### Structural characteristics of the RSI proteins and candidate orthologs

**Fig 1:**
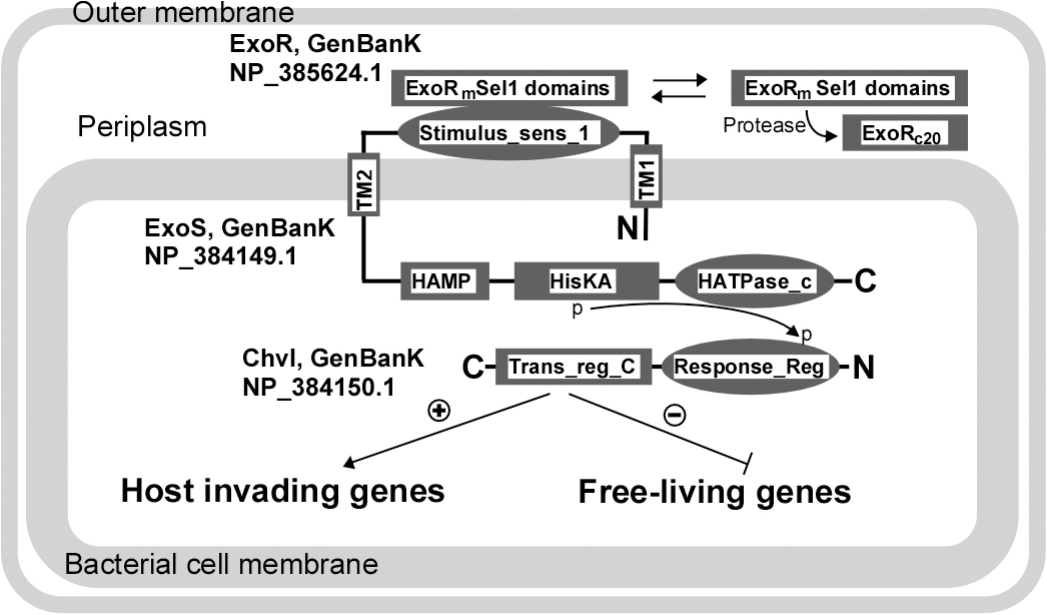
ExoR, ExoS, and ChvI proteins interact to create the RSI Invasion Switch in *S*. *meliloti*. ExoR is composed of Sel1 repeats, is secreted to the periplasm as ExoR_m_ (lacking the signal peptide), and is cleaved to release its suppression of ExoS. The domain architectures of ExoS and ChvI, along with phosphorylation sites, are illustrated here. Signal transmission is achieved via a phosphorylation cascade and ChvI completes the “switch” by binding to DNA to alter gene expression [96]. Our current model suggests that the levels of ExoR_m_ in the periplasm are maintained through the combination of biosynthesis and proteolysis. Proteolysis is possibly sensitive to host signals or to changes in environmental conditions. These mechanisms allow for (1) ExoR_m_ levels to be dramatically reduced in the presence of host signals, (2) the turning “ON” of the RSI invasion switch, (3) the activation of host-invading genes, and (4) the suppression of free-living genes.

*S*. *meliloti* Rm1021 ExoR (GenBank Accession NP_385624.1) is a periplasmic protein of 268 residues with a signal peptide and a set of six Sel1 repeats [17] (Fig 1). These structural characteristics determine the function of ExoR within the invasion switch, and we identified the sequences that could potentially fulfill similar functions. Thus, we further narrowed our putative ExoR orthologs by requiring consensus predictions for both secretion and Sel1 domain repeats.

The majority of the 57 candidate ExoR orthologs (91%) were less than 275 residues, while five encoded more than 350 residues (from *Azorhizobium caulinodans* ORS 571, *Hyphomicrobium denitrificans* ATCC 51888, *Novosphingobium aromaticivorans* DSM12444, *Pelagibacterium halotolerans* B2, *Rhodomicrobium vannielii* ATCC17100) (Table 1 and S2 Table). Outlying sequence-encoded structural features among ortholog candidates, as compared to Rm1021 ExoR, included localization signatures and non-Sel1 domains. Four candidate ExoR orthologs unexpectedly returned strong predictions of non-periplasmic localizations, i.e., lack of signal peptide, potential transmembrane helices (TMH), and/or a lack of other secretion mechanisms (from *A*. *caulinodans* ORS 571, *Beijerinckia indica* ATCC 9039, *Parvibaculum lavamentivorans* DS-1, and *P*. *halotolerans* B2) (Table 1 and S2 Table). Based on current understanding of RSI, proteins that are not secreted to the periplasm cannot function as ExoR-like environmental sensors. One candidate sequence was found to encode a domain other than Sel1 (Sporulation related domain (SPOR), Pfam: PF05036R; *Novosphingobium aromaticivorans*, Table 1 and S2 Table). The final set of 47 candidate ExoR orthologs retained after these analyses were found predominantly in Rhizobiales, and have repeats of a binding domain (Sel1) in addition to consensus predictions for secretion.

All identified ExoS and ChvI orthologs have high levels of sequence similarity (S1 Table) and domain architectures that mirror the *S*. *meliloti* ExoS and ChvI TCS. These conserved structural characteristics suggest that ExoS and ChvI orthologs may respond to external stimuli and activate transcriptional responses similar to those of *S*. *meliloti* and *A*. *tumefaciens*. In our searches, we found ExoS and ChvI orthologs in every genome that encoded an ExoR ortholog, the majority of which were found among the Rhizobiales (Table 1 and S1–S3 Tables).

### Phylogenies of RSI proteins are consistent with each other and with accepted Rhizobiales phylogeny

The TCoffee Espresso [76] multiple sequence alignment (MSA) for the ExoR, ExoS, and ChvI ortholog sets required little manual adjustment and supported our domain predictions (S1 Fig). The predicted Sel1 domains of the ExoR orthologs aligned well with few notable insertions. The ExoS and ChvI MSAs also supported domain annotations and predictions.

**Fig 2:**
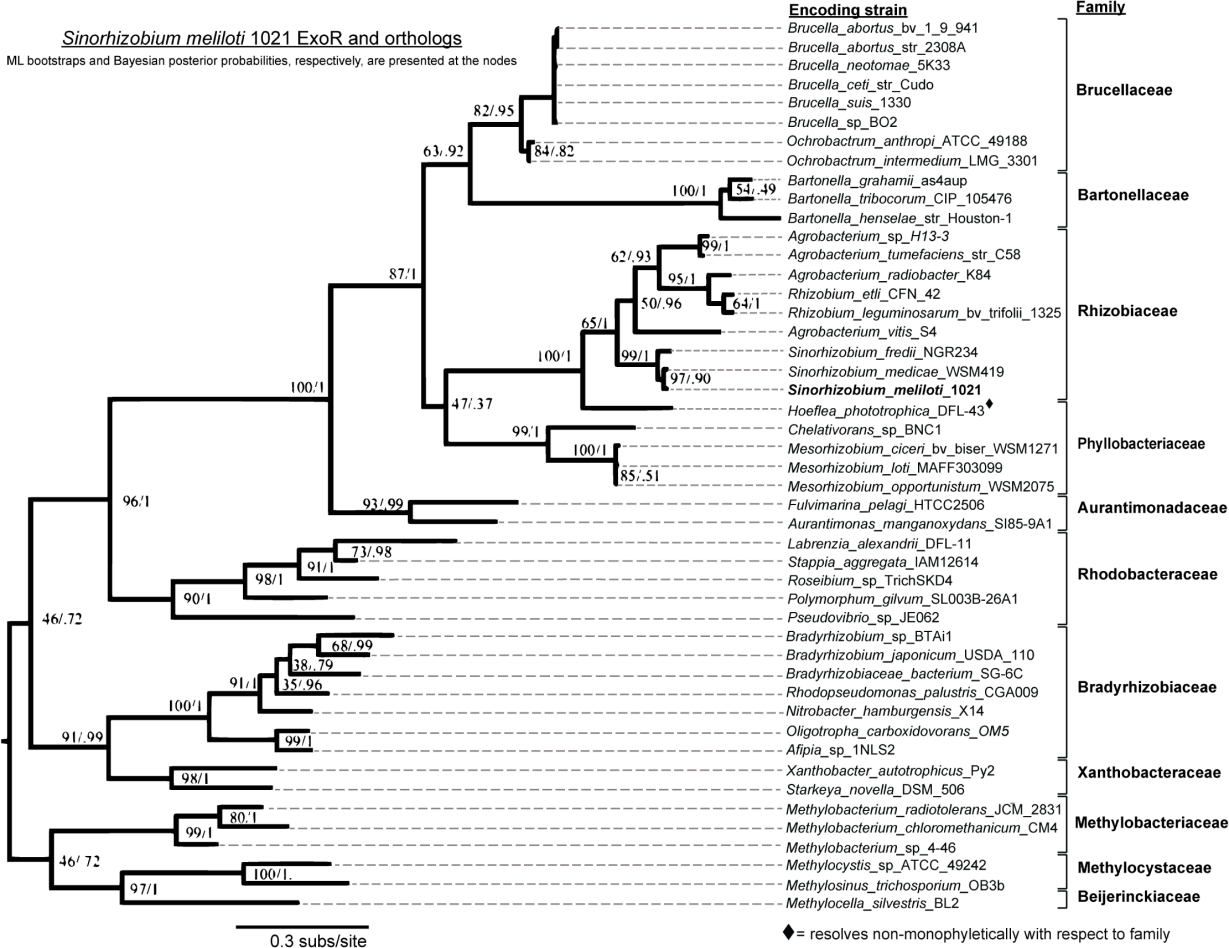
Evolutionary history of ExoR is congruent with speciation history of Rhizobiales. ML and Bayesian phylogenetic patterns support the existence of an ExoR ancestor that arose prior to diversification among the *Rhizobiales* and five *Rhodobacterales* species identified in our genomic searches. The ExoR gene was maintained among these species, giving rise to the ortholog set. Phylogenetic reconstruction of ExoR orthologs agrees with currently accepted speciation patterns.

**Fig 3:**
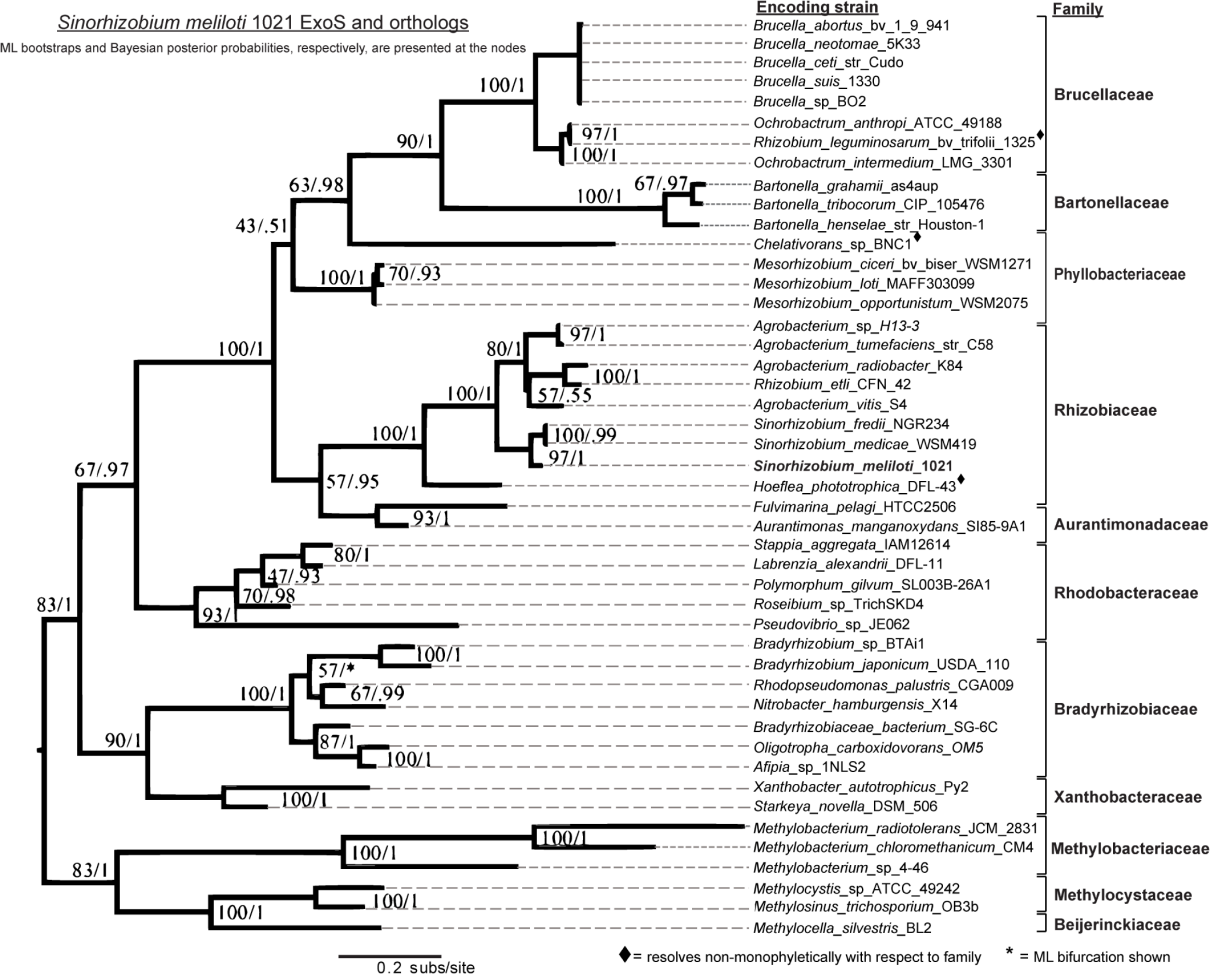
Concordant evolution of ExoS and ExoR. The overall evolutionary rates and branching pattern of the ExoS phylogeny agree with those of ExoR (see S3 Fig for mirrored comparisons). Although not all ExoS orthologs resolve strictly with family speciation patterns, most differences are minor. The *Phyllobacteriaceae* orthologs are, in fact, the most significant difference in the predicted histories of the ExoR and ExoS sets. The comparison of the ExoS reconstruction to the ExoR (Fig 2) supports the assertion that the evolution of the two ortholog sets was concordant.

**Fig 4:**
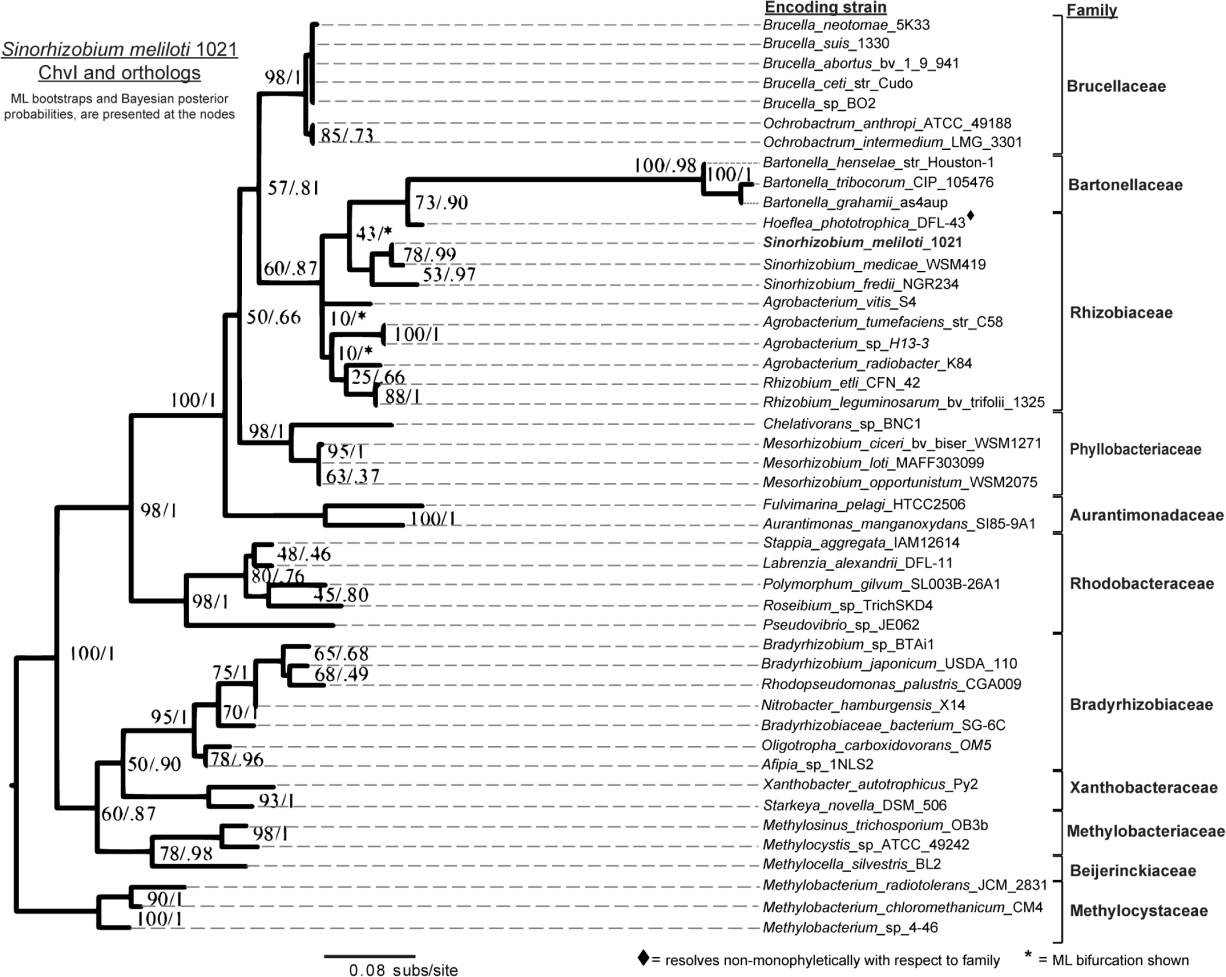
Conserved sequence evolution of ChvI in Rhizobiales and selected Rhodobacterales. As a cognate TCS pair, ChvI and ExoS sequences are expected to diversify at similar rates. However, the ChvI phylogeny suggests that it is under strong purifying selection with the lowest amino-acid substitution rate among the three components. It is possible that the functional role of ChvI orthologs (i.e., DNA binding) limits diversification.

Well-supported protein phylogenies (Figs 2–4) were produced for the ortholog sets after non-resolvable sequences from five taxa were excluded. These outliers were identified by their comparatively long branch lengths in a preliminary ExoR phylogeny that was poorly supported at numerous nodes (S2 Fig). Three of these five outlying sequences had predictions of divergent localizations (*Ahrensia* sp. R2A130, *Magnetospirillum magnetotacticum* MS-1, *Rhodomicrobium vannielii* ATC17100), while the other two protein sequences are notably longer than ExoR (*Hyphomicrobium denitrificans* ATCC 5188, *Hyphomicrobium* sp. MC1) (Table 1 and’S2 Table). Given the exclusion of these sequences, the overall ExoR reconstruction bootstrap support was highly improved (Fig 2 versus S2 Fig), particularly at basal bifurcations.

In the final ExoR trees (Fig 2), a node that divides the *Rhizobiaceae* from the *Phyllobacteriaceae* is not well supported by either the likelihood or Bayesian method, but is similar to speciation patterns (PATRIC Rhizobiales phylogeny, http://patricbrc.vbi.vt.edu/ [92,93]). The ExoS reconstructions (i.e., ML and Bayesian) agreed in 39 of the 40 bipartitions (98%) (Fig 3). In the ChvI ortholog reconstructions, the Bayesian tree was better supported than the ML in 68% of the nodes (Fig 4). The most significant variation between the two methods in the ChvI trees was found within the *Sinorhizobium* spp. (Fig 4) and the divergence of the *Phyllobacteriaceae* from the *Rhizobium/Bartonellaceae* clade was unresolved in all of the RSI ortholog trees.

The lower level support in the ChvI reconstruction is likely due to low sequence divergence since ChvI-like proteins are extremely common as bacterial transcription factors. The production of high-quality alignment blocks removed much of the original sequence variation present in our ortholog sets. The lack of resolution among *Brucellaceae* RSI orthologs is most likely also due to low genetic, and thus sequence, divergence [97]. For all RSI tree comparisons, we have considered the *Brucella* species proteins as a unified group.

The three sets of ortholog trees were compared pairwise (S3 Fig) to assess the possibility of their synchronized evolution. The similar (73% of family nodes) branching patterns of the ExoR and ExoS ortholog trees (Figs 2 and 3), as well as comparable mutation rates (0.2 and 0.18, respectively) suggest similar evolutionary pressures. Surprisingly, the ExoS-ChvI ortholog tree pair demonstrates fewer congruent (45%, or 5 of 11) family nodes (S3 Fig), even though linked evolutionary pressure in TCS protein pairs is well-established. Since the mutation rate in the ChvI ortholog protein set is low (approximately 25%) in comparison to those of both the ExoR and ExoS orthologs, we propose that purifying pressures lead to high levels of conservation and low mutability among the ChvI othologs. While our comparison of the ExoR-ExoS ortholog trees suggests co-evolution, the initial resolution of the trees, stochasticity, non-monophyletic groups, and divergence in the more ancient lineages must be taken into account. In summary, the phylogenies of the three RSI proteins (Figs 2–4) are highly consistent, not only to each other, but also to the known phylogeny among these Alphaproteobacteria species.

### A single evolutionary origin and the persistence of RSI switch in Rhizobiales

**Fig 5:**
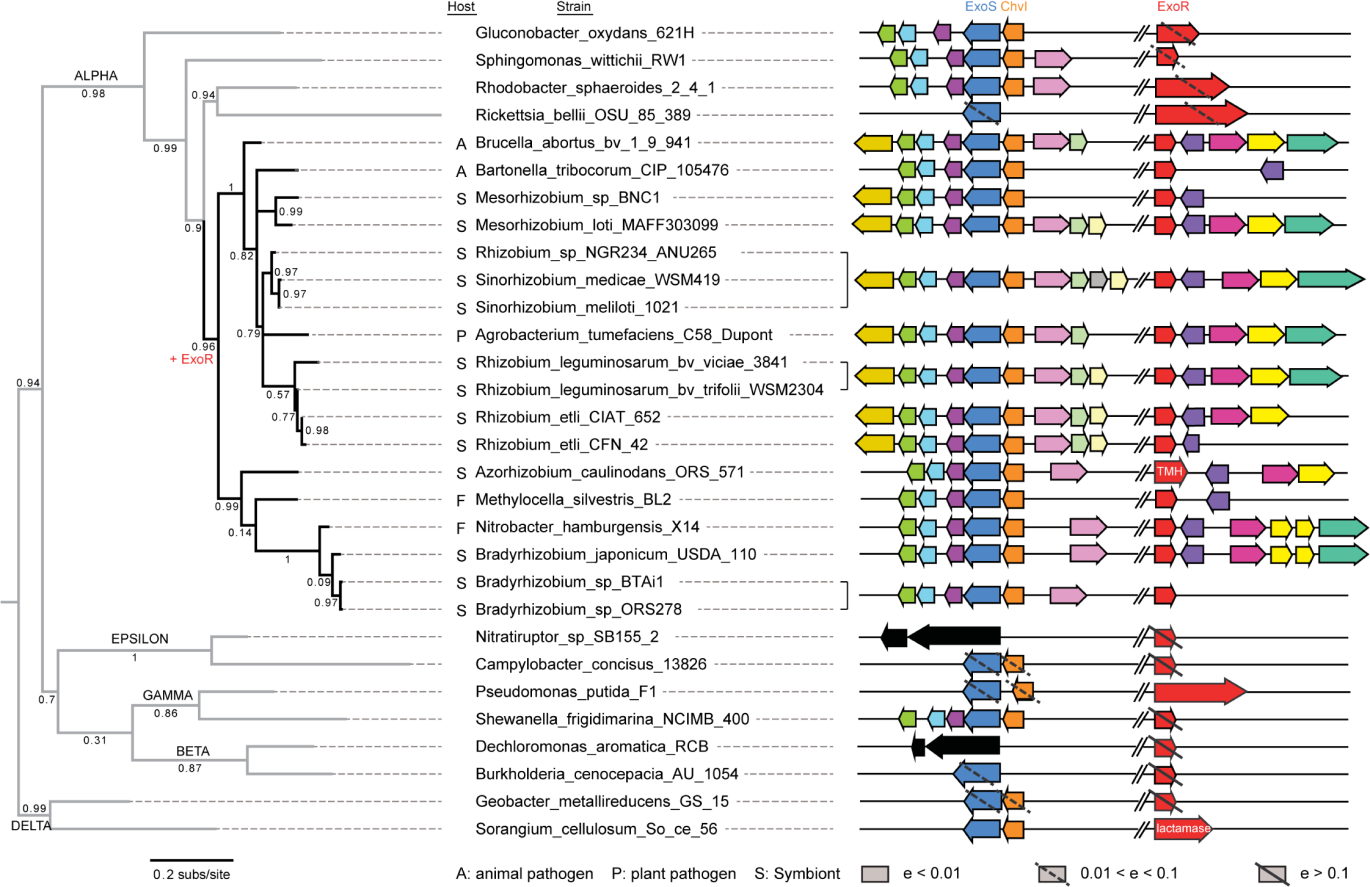
RSI Synteny analysis within Rhizobiales and ancestral Alphaproteobacteria. Clusters of orthologous gene (COG) groups for representative Proteobacteria are presented for comparison to the prototype, the loci of Rm1021. Each COG, as defined in the IMG database, is represented by an arrow of different color. For the *exoR-exoS-chvI* genes, a lack of homologs or orthologs is presented as an ORF with a solid or dotted slash, respectively. The sizes and positions of the ORFs are approximate, representing relative expansions/deletions. The ExoR ortholog found in *Azorhizobium caulinodans*, although annotated as an exopolysaccharide regulator, is predicted to encode transmembrane helices and is a structural outlier as compared to ExoR-like molecules found in other Rhizobiales. Lifestyles and host type are given by A (animal pathogen), P (plant pathogen), or S (symbionts). Gene loss/gain is indicated with-/+, respectively. COG# color key (L to R): Dark yellow, 0499; green, 1925; light blue, 2893; dark violet, 1493; blue (exoS/chvG), 0642; orange (chvI), 0745; light violet, 1866; pale green, 1186; grey, 1652; white, 3145; red (exoR), 0790; purple, 0708; magenta, 1502; bright yellow, 0232; turquoise (sporulation-domain encoding). In Rm1021 RSI loci are *exoS* (bp49252–51039), *chvI* (51419–52141), and *exoR* (1637310–1638116) on the SMc chromosome.

To assess RSI conservation and loci architectures both within and outside of the Rhizobiales, we conducted synteny analysis (Fig 5). Orthologous RSI pathways were mapped onto the Alphaproteobacterial phylogeny with gene neighborhoods organized according to species divergence to visualize (1) gene losses/gains and (2) patterns of co-evolution [95].

ExoR orthologs were not identified in a majority of species within any Alphaproteobacterial order other than Rhizobiales. Conversely, the presence of ExoS/ChvI orthologs is an ancestral state to the order (Fig 5). The evolutionary pathway to this state is not conserved as ExoS/ChvI orthologs were identified, but rarely, in the other Proteobacterial classes (e.g., *Sorangium cellulosum*, Deltaproteobacteria). Sequences with distant relationships to ExoR (BLASTs E<10e-5, 75% alignment), were identified outside of Rhizobiales, but uniformly lacked best-best character with respect to Rm1021 ExoR and its crucial structural characteristics (S2 Table). Fully orthologous RSI-like protein sets were identified only within Rhizobiales as a taxonomically conserved characteristic, suggesting that this pathway is a lineage-specific adaptive feature of the order.

Among the sampled genomes, the ExoR ortholog neighborhoods (COG0790) show low levels of conservation in comparison to the ExoS/ChvI orthologs. Although the COG 0232 (dGTP triphosphohydrolase) and COG 0018 (arginyl-tRNA synthetase) groups are commonly found with the ExoR COG 0790, there are genomes illustrated in Fig 5 that do not follow this pattern (e.g, *Mesorhizobium etli* CFN42 and *Mesorhizobium sp*. BNC1). COG 0708 (exodeoxyribonuclease III), is the only COG class within which the ExoR orthologs are consistently located. However, since COG 0708 and ExoR orthologs are encoded on opposite strands, it is unlikely that they are under similar transcriptional control.

The ExoS (COG0745)/ChvI (COG0642) ortholog loci show notable conservation which extends into more ancestral orders such as the Rhodobacterales. The phosphotransferase/HPr related cluster of COG 1493, 2893, and 1925 (incomplete phosphotransferase system among the Alphaproteobacteria), along with *exoS* constitute an operon in Rm1021. This architecture is conserved among the Rhizobiales and suggests that the operon itself is conserved.

### Non-orthologous ExoR proteins may serve divergent functions in non-Rhizobales species

Of the 35 sequences identified as potential Rm1021 ExoR homologs, but not as orthologs (Table 1 and S2 Table), approximately one-third were found to have divergent structural and/or functional predictions. Outlying structural predictions included the SPOR (Pfam: PF05036), PG_binding_1 (Pfam: PF01471), and Peptidase_C14 (Pfam: PF00656) domains. Unexpected, predicted functionalities included beta-lactamase activity and organelle localization signaling (e.g., PodJL). These putative ExoR homologs with alternative structural and/or functional predictions were found in more ancestral lineages, such as the Caulobacterales, the Rhodobacterales, the Rhodospirillales, and the Sphingomonadales (S2 Table). SPOR domains and PodJL proteins are associated with peptidoglycan binding. Both the putative beta-lactamase and the peptidase C14 have associated binding predictions. We conclude from gene neighborhood syteny analysis (Fig 5) that there has been a single evolutionary origin for the complete set of RSI switch orthologs in a recent common ancestor of Rhizobiales. The RSI Switch has persisted throughout the subsequent divergence within the order.

### Evolutionary variability within ExoR and ExoS sensing domain

**Fig 6:**
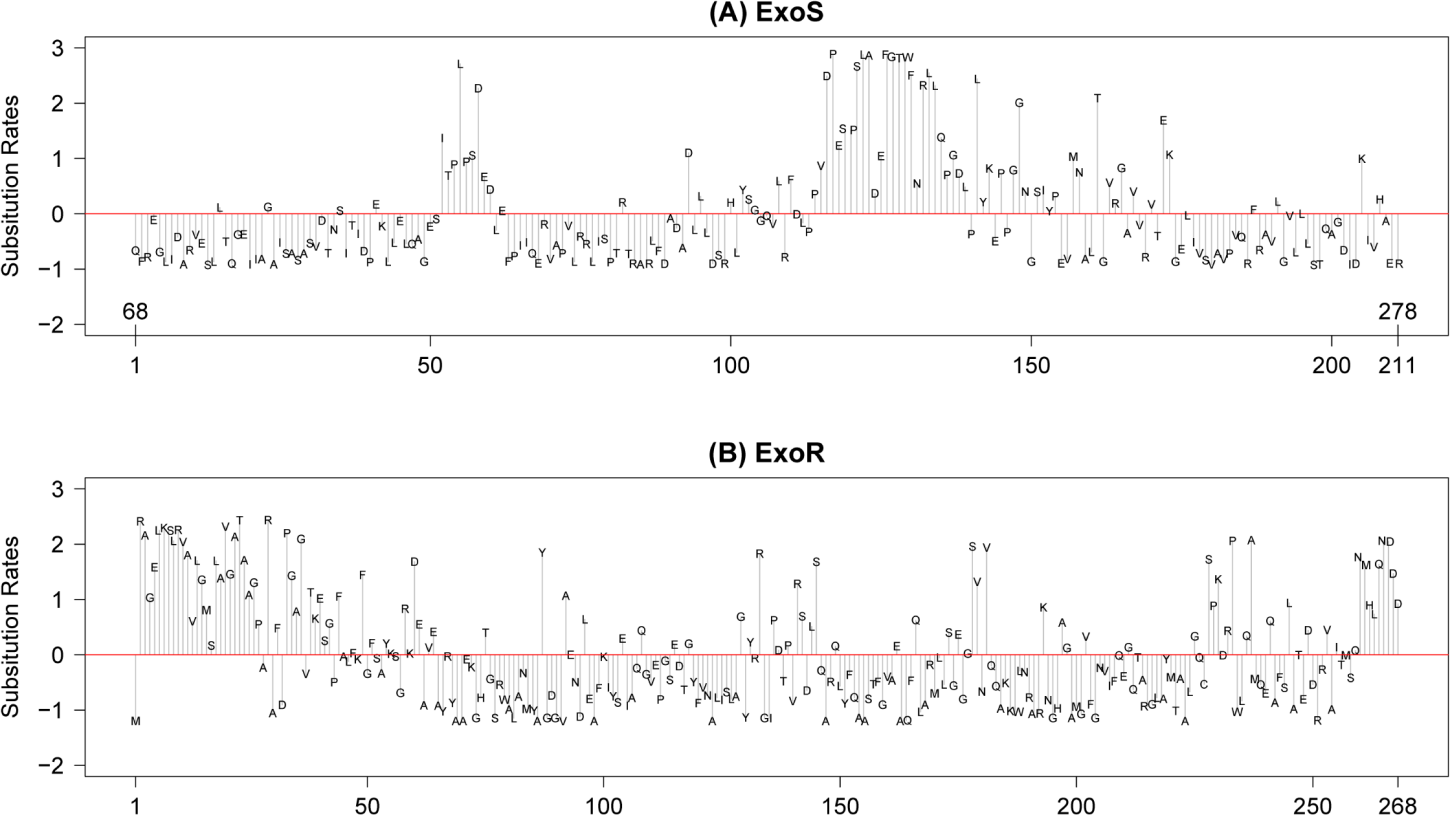
Site-specific evolutionary rates of ExoR (A) and ExoS (B). Substitution rates (y-axis) at amino-acid positions (x-axis, numbers based on (A) the periplasmic region of *S*. *meliloti* ExoS and (B) full-length ExoR) were obtained using Rate4Site [94]. While variable and conserved residues are evenly dispersed in ExoR (B), two variable residues are notable and specific to two regions in ExoS (A). We hypothesize, subject to experimental verification, that these two variable regions form binding domains to ExoR and are the molecular basis of host-specific responses among Rhizobiales species.

ExoR orthologs evolve faster than those of ExoS, with overall average pairwise sequence identities of 44.6% and 61.4%, respectively, based on comparative alignments among 46 non-redundant orthologs from Rhizobiales species. The N-terminus and C-terminus of ExoR are the most variable; although, conserved and variable residues are dispersed throughout the molecule (Fig 6B). In contrast, sequence variability of ExoS is concentrated in two regions, one short region consisting of the sequence ITPLPSDED (residues #52–60 of periplasmic region/#119–127 of full ExoS) and another longer regions consisting of PVDPESPSLADEFGTWFNRLLQPGDL (#114–139 of periplasmic/#181–206 full ExoS) (Fig 6A).

**Fig 7:**
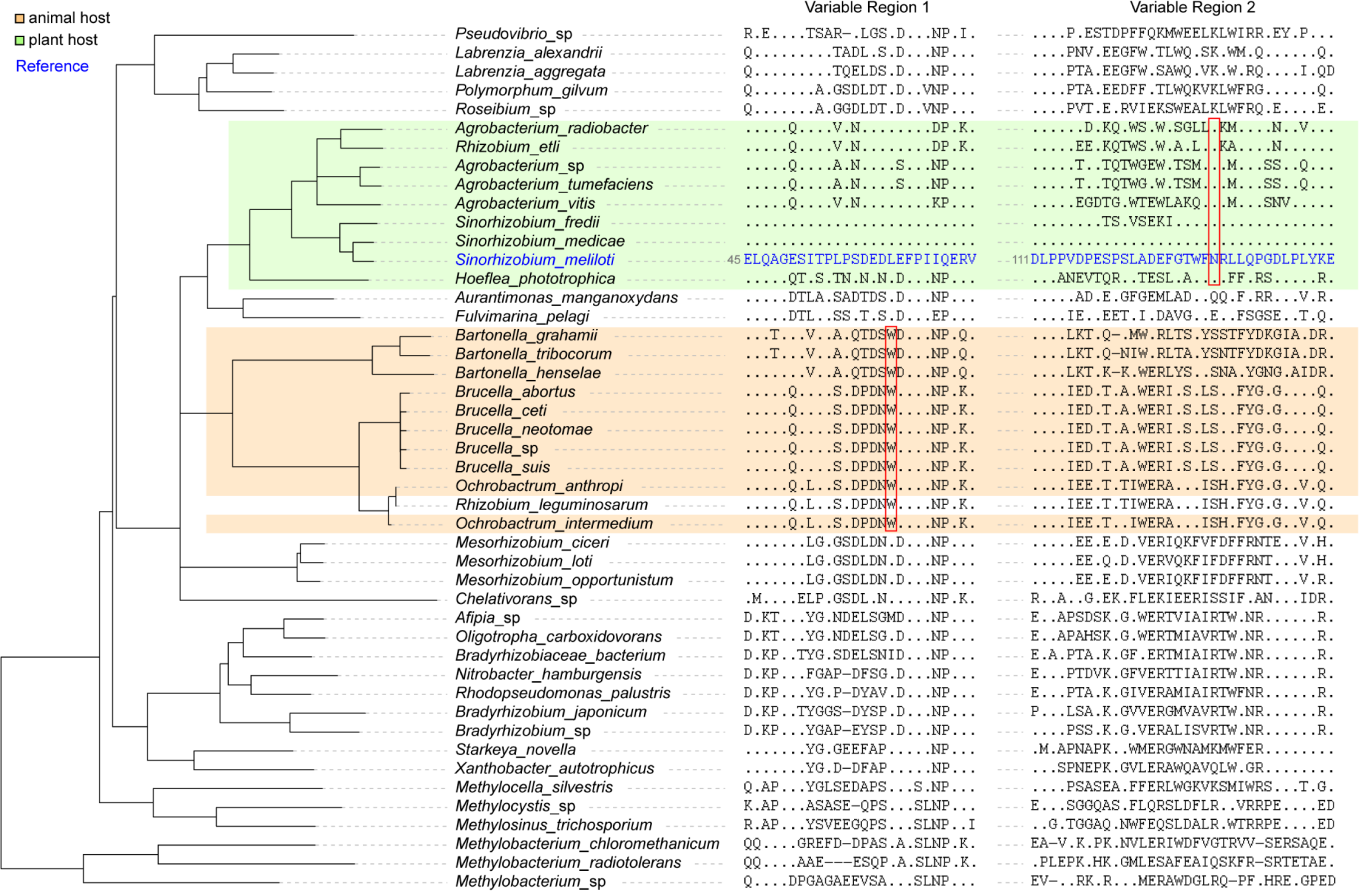
Two host-associated radical amino-acid substitutions in ExoS. Sequences within the two hypervariable regions (Fig 6) of ExoS are displayed according to a phylogeny of ExoS sequences. Based on parsimony reasoning, an asparagine residue (N131of the ExoS periplasmic region) is an evolutionarily derived state that is conserved among plant- or dinoflagellate-associated taxa. A tryptophan residue (W61of the ExoS periplasmic region) is derived from a leucine ancestor and conserved among animal-infecting species with the exception of *Rhizobium leguminosarum* (a plant pathogen).

Further evolutionary reconstruction based on parsimony revealed differential lineage-specific amino-acid substitutions associated with the emergence of species using animals as host versus plants (Fig 7). In the N-terminal hypervariable region of ExoS, a tryptophan, derived from an otherwise highly conserved leucine, is found among the *Brucellaceae* orthologs, along with the *Rhizobium leguminosarum* sequence. Although the plant symbiont *R*. *leguminosarum* is an outlier, the other species (*Brucellaceae* and *Bartonellaceae*) that encode tryptophans at this site are intra-erythrocyte, animal-infecting pathogens. The plant-associated *Rhizobiaceae* resolved together according to parsimony and consistently encode an asparagine within the more C-terminal ExoS hypervariable region (Fig 7). *Hoeflea phototrophica*, a species that has been found to associate with dinoflagellates [98,99], is included in this group, suggesting that a signaling commonality may exist between plant and protist host invasions.

## Discussion

The RSI invasion switch regulates biochemical changes within *Sinorhizobium meliloti* to produce invasion-competent bacteria from free-living forms, a morphogenesis that is necessary for host entry and the establishment of symbiosis [11,12,100]. The genomic encoding of RSI orthologs has been correlated to organisms that significantly impact human health and commerce, including diazotrophs and both animal and plant pathogens [30,34,35,36,37]. As detailed in the result section and following discussion, our analyses suggest that ExoR-ExoS-ChvI-like pathways exist as coevolutionary conserved units of signaling function, and they represent a molecular signature within a specific phylogenomic range. Most importantly, our results show that the RSI invasion switch could function in at least 47 different species which include mammalian and plant pathogens.

### The inferred evolutionary history of the RSI switch

TCS proteins similar to ExoS and ChvI are found in all domains of life [101] and their gains can be adaptive within new environments [95]. The conservation patterns of the RSI orthologs found here suggest that, in this case, a third protein was gained, adding to an existing TCS. Although ExoS- and ChvI-like proteins were found at the base of Alphaproteobacteria, ExoR orthologs were not (e.g., *Novophingomondaceae* and *Rickettsiaceae*).

RSI ortholog loci patterns are consistent across the taxa investigated here. However, *exoS-* and *chvI*-like genes are adjacent while *exoR*-like loci are separate indicating that a transfer event of all three genes as a functional unit likely did not occur. Lineage expansion and domain shuffling [95] is a known diversification process in TCSs and could possibly extend to a third, interacting protein. Five Rhodobacterales (ancestral to Rhizobiales) were found to encode ExoR orthologs, but seven other Rhodobacterales species encode ExoR-like proteins with alternate functional and structural predictions. These sequences were selected from our BLASTs as putative homologs, but failed to satisfy our ortholog criteria.

In the prototypical *S*. *meliloti* RSI system, ExoR is composed solely of Sel1 domains, interspersed with linking regions [17]. In contrast, putative ExoR homologs (but not orthologs) in families basal to the Rhizobiales encode alternative and additional domains such as SPOR, PG_binding_1, and C14_peptidase. Notably, SPOR and PG_binding_1 domains are associated with the periplasm and binding within this cellular compartment. Such ancestral functional profiles suggest that RSI-like modes of interaction (a negative regulator that binds a kinase sensing domain) may have specialized from other examples of periplasmic binding, potentially via domain loss, to provide environmental response and transcriptional switching. These findings suggest a diversification event for the gain of ExoR-like orthologs within the orders ancestral to Rhizobiales. Changes within a third protein, such as an externally responsive sensor like ExoR, may have improved the necessary fitness to a Rhizobiales ancestor for adaptation to a new niche, such as nitrogen fixation, host-specific intracellular habitation, or alternative nutritional opportunities.

### Facultative lifestyles are characteristic of organisms that encode RSI Switch orthologs

We have found that tripartite RSI orthologous gene sets are characteristic of Rhizobiales, an order that includes numerous pathogens and symbionts. While lineage-specific presence of any one gene is itself an indication of species adaptation, phylogenetic co-occurrence of all three interacting partners strongly suggests that the RSI switch is a key adaptive pathway in Rhizobiales. Of the multiple RSI switch-encoding bacteria that can reside intracellularly within their hosts, the majority are predominantly facultative rather than obligate. Gross transcriptional switching is consistent with proposed RSI functions, and may regulate opportunistic responses to environmental signals by coordinating facultative changes in metabolism, membrane chemistry, and motility within alternate environments. Of the RSI-encoding species that lack known hosts we suggest that those with unstable genomes may be under selection for host association, such as select *Brucella* spp., perhaps due an advantage in their a free-living state.

Many facultative organisms, both diazotrophs and well-known pathogens, are found in the Rhizobiales. These species switch to intracellular lifestyles when enabled by evasion of, or resistance to, their hosts’ immune responses. Accordingly, obligate intracellular pathogens, such as the well-known *Rickettsia* spp., were not found to encode RSI orthologs even though species from their sister clades were identified. S3 Table presents the lifestyles, hosts, and significant roles of the taxa included in this investigation. More than half of the listed species associate facultatively with other organisms and most frequently in symbiotic or pathogenic relationships. Many of the remaining species utilize or oxidize unique carbon, nitrogen, and metallo-organic molecules. Signaling links between their RSI-like switching pathways and these unusual metabolic pathways are not currently known, but are worthy of investigation.

Our phylogenetic analyses suggest that ExoS is under independent, site-specific positive pressures associated with host-type (Fig 7). As seen in ML/Bayesian phylogenies (Figs 2–4), the emerging pathogen *Bartonellaceae* are noticeably under more diversifying pressure than the orthologs from other families of the Rhizobiales order. Other emerging mammalian pathogens were identified with RSI sets (*Brucella* spp., *Methylobacterium radiotolerans*, and *Ochrobactrum* spp.) and outnumber the plant pathogens investigated here (*Agrobacterium* spp.). Organisms with unique facultative metabolisms (e.g., methanotrophism), that may be under switch-like transcriptional control, make up the majority of the non-pathogenic species that encode RSI-like pathways.

Closer investigation of ExoS demonstrates that the most rapidly evolving regions of ExoS are within the periplasmic regions and that characteristic insertions and radical substitutions are present (Figs 6 and 7). We have uncovered specific sequence motifs that correlate with host type (i.e., plant versus animal) among many of the species investigated here. These results not only provide impetus for functional testing via RSI mutagenesis, but they also suggest that adaptive pressures have acted on switch proteins in plant invading-species, animal pathogens, and other classes of less characterized species that invade basal eukaryotic hosts such as protists.

### The implications of an RSI invasion switch within pathogenic species

*S*. *meliloti* ExoR mutants show reduced invasion efficiency of their host plant, alfalfa [12,27,28,100]. ExoS/ChvI homolog mutants have also been correlated to reduced invasion abilities [14,34,36,37]. Given the high incidence of facultative intracellular lifestyles within the Rhizobiales, and the genetic similarity of pathogenicity and symbiosis islands, a shared sensing mechanism for both types of invasion is possible. Responding in an opportunistic way to specific external conditions enables both pathogenic and symbiontic lifestyles. RSI switch conservation in both symbionts like *S*. *meliloti* and pathogens like *Brucella* spp. suggests that the pathway which is mostly understood for its contribution to symbiosis, may also contribute to pathogen success.

In addition to the RSI system, other TCSs have evolved with accessory proteins, or adaptors, that control the functional TCS status based on environmental conditions [102]. The CpxP-CpxA-CpxR system [103] is conserved across a phylogenetically broad group of bacteria which includes Gram negative species such as *E*. *coli*. In this system, CpxP directly binds the sensor kinase CpxA under normal physiological conditions, but becomes sensitive to proteolysis in the presence of misfolded proteins. CpxP cleavage relieves its suppression of CpxA, a sensor kinase, which allows *E*. *coli* cells to respond to environmental stresses by increasing levels of chaperones and trafficking factors [103]. In the *Streptomyces* HpbS-SenS-SenR system, HpbS releases its negative regulation SenS through a conformational shift under high oxidative stress [104]. The induction of the *Streptomyces* SenS-SenR TCS leads to an increase in expression of protective anti-oxidative catabolic enzymes, like peroxidases and catalases [104]. Given the restricted and unified nature of the RSI orthology group, in addition to our experimental understanding of RSI, it is possible that the RSI system functions under similar environmental control and activation as these to tripartite signaling systems.

Orthologs of the RSI invasion switch show synteny and highly similar histories within Rhizobiales. Our results suggest that these RSI-like orthologs sets have potentially experienced unified evolutionary histories and have responded to similar pressures to provide a key evolutionary innovation (KEI) for these species. Experimental works [12,13,15,16,18,31] support the necessity of RSI within Rhizobiales. Our phylogenomic RSI analyses provide an informed starting point not only to characterize host entry in emerging pathogens, but to study a potential connection between mechanisms of host entry in both symbiosis and pathogenesis. By determining the taxonomic range of this “switch,” we have facilitated the targeting of experimental analyses. By delineating potential conservation in invasion signaling, research aimed at pathogens may be facilitated in non-pathogenic species, creating safer short- and long-term research environments.

## Supporting Information

S1 Fig Characteristic domains of the ExoR homolog/ortholog data set. Alignment has been trimmed at both termini and colored to show putative Sel1 domains. The longest putative homolog (RhodomicV) included was trimmed as follows: 49 and 101 residues from its N- and C-terimini, respectively. 5 sequences were removed from this set of 52 for the final reconstructions and analyses (see S3 Fig). Naming conventions are given in S1 Table.
(PDF)

S2 Fig Preliminary ExoR ML reconstruction built with 52 species. Bootstrap values are given. Ahrensia, HypmcrDn, HypmcrSp, RhodomicV, and MgntspirM were considered to be non-resolved. Main text Fig 2 is built without these taxa. Color code: *Brucellaceae*, red; *Bartonellaceae*, light green; *Phyllobacteriaceae*, pink; *Rhizobiaceae*, royal blue; *Aurantimonadaceae*, orange; *Rhodobacteraceae*, dark purple; *Bradyrhizobiaceae*, light blue; *Xanthobacteraceae*, yellow; *Methylobacteriaceae*, coral; *Methylocystaceae*, dark green; *Beijerinckiaceae*, brown. Naming abbreviations given in S1 Table.
(TIF)

S3 Fig Mirrored comparisons: ExoR-ExoS and ExoS-ChvI paired ortholog phylogenies. (ExoR orthologs on left ExoS on right, first figure; ExoS orthologs on left and ChvI on right, second figure) Similar branching patterns among the RSI switch proteins suggest a unified phylogenetic history. Bootstrap values, followed by posterior probabilities, are given. Starred nodes are non-congruent. Color codes given in S2 Fig.
(PDF)

S1 Table RSI ortholog information. GenBank accession numbers & BLAST results.
(PDF)

S2 Table ExoR ortholog analyses. Results of from reciprocal BLASTs and predictions of signal peptides, domains, and transmembrane helices (TMH).
(PDF)

S3 Table Phenotypes for species demonstrating RSI co-occurrence. Every Alphaproteobacterial genome with an identified ExoR ortholog also encodes ExoS and ChvI orthologs. Many of these species are facultatively intracellular and include well-known examples of both pathogens and symbionts.
(PDF)
