# Supplementary figures and images for: Phylogenetic Co-Occurrence of ExoR, ExoS, and ChvI, Components of the RSI Bacterial Invasion Switch, Suggests a Key Adaptive Mechanism Regulating the Transition between Free-Living and Host-Invading Phases in Rhizobiales
Source: PLoS One. 2015 Aug 26;10(8):e0135655. doi: 10.1371/journal.pone.0135655 (PMC4550343; doi:10.1371/journal.pone.0135655)

10 20 30 40 50 60 70 80 90

[illegible]

**Supplemental S1 Fig., continued.**

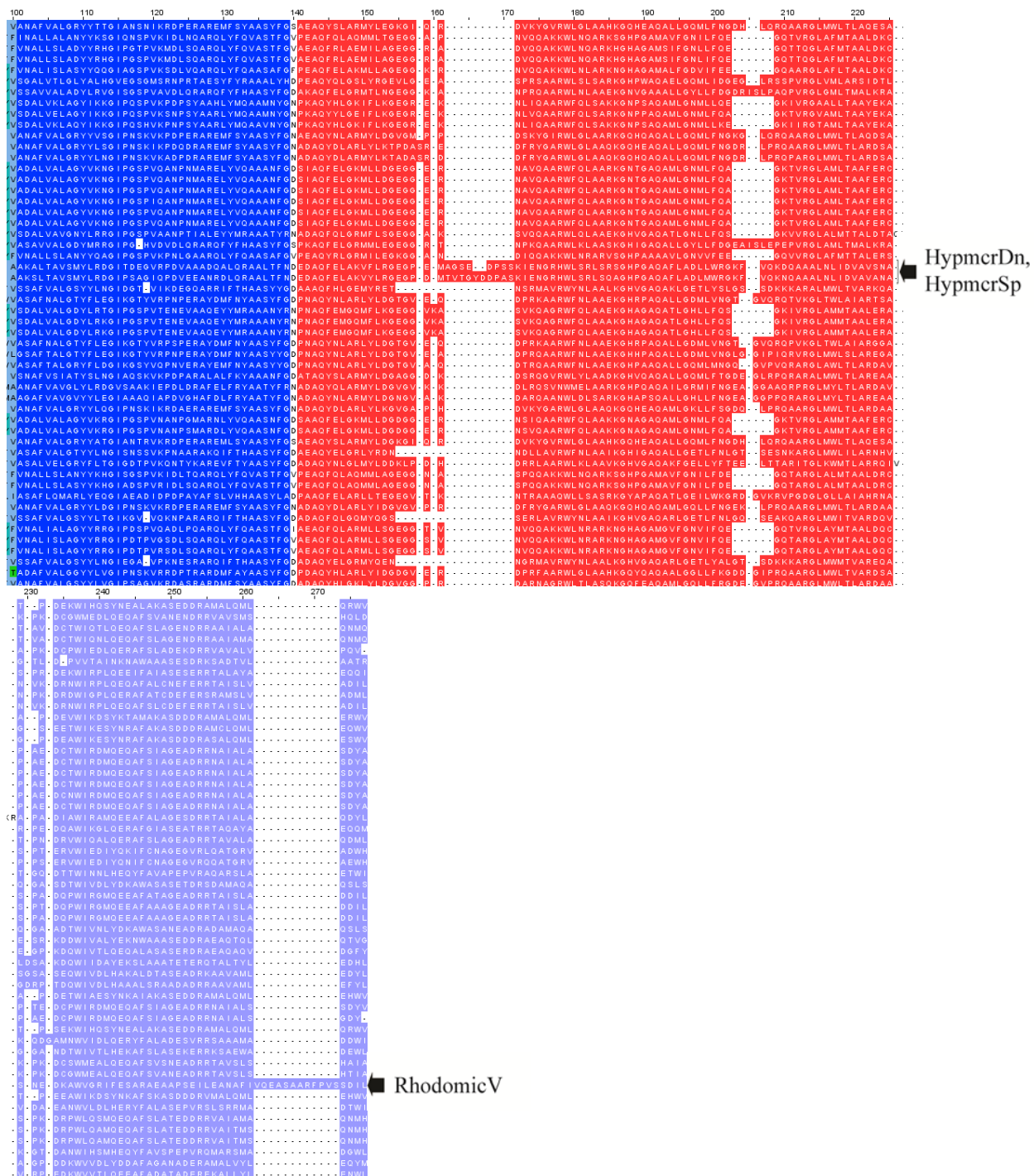

Supplement: S1 Fig — Alignment has been trimmed at both termini and colored to show putative Sel1 domains. The longest putative homolog (RhodomicV) included was trimmed as follows: 49 and 101 residues from its N- and C-terimini, respectively. 5 sequences were removed from this set of 52 for the final reconstructions and analyses (see S3 Fig). Naming conventions are given in S1 Table. (PDF) [file pone.0135655.s001.pdf]

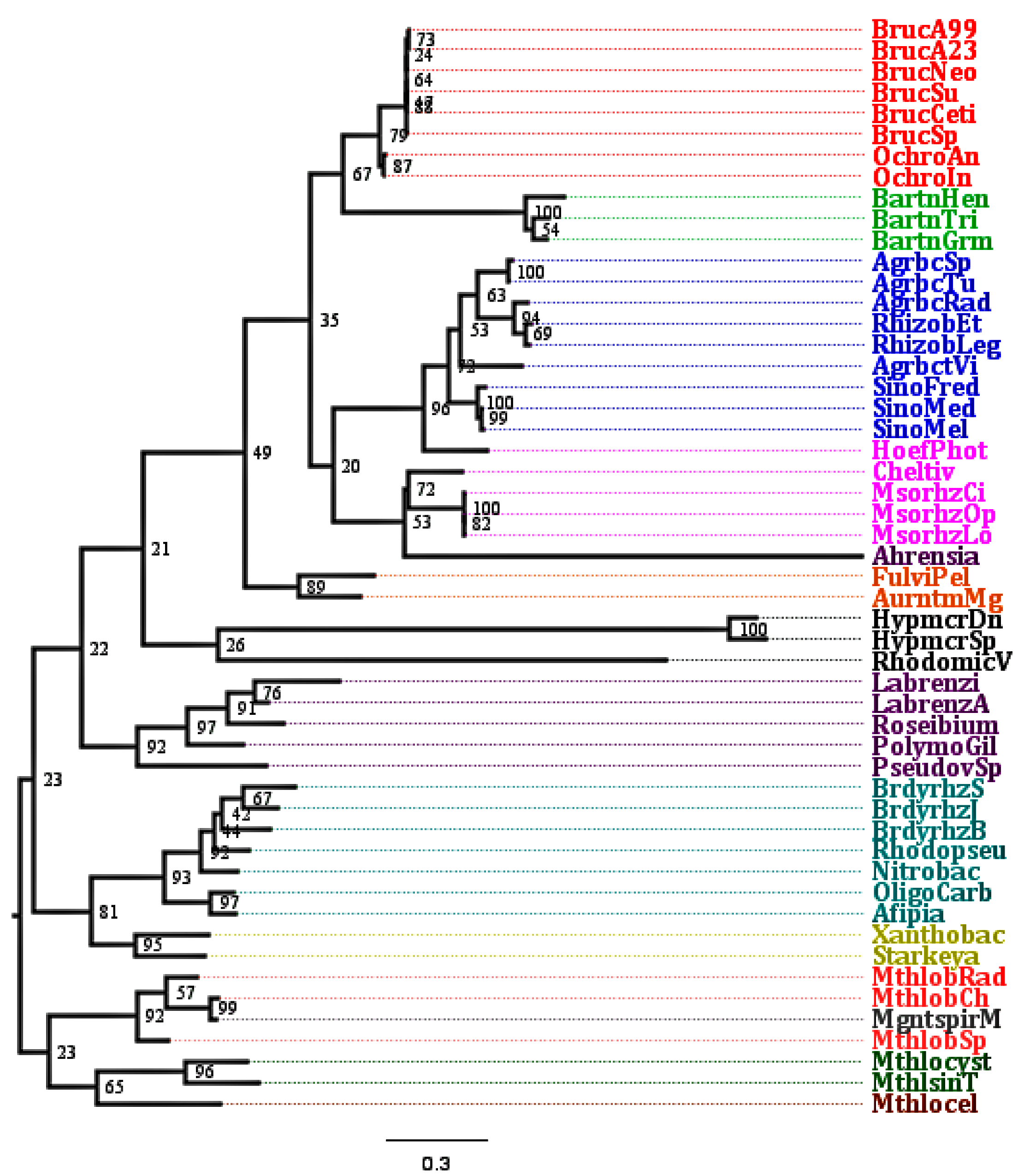

Supplement: S2 Fig — Bootstrap values are given. Ahrensia, HypmcrDn, HypmcrSp, RhodomicV, and MgntspirM were considered to be non-resolved. Main text Fig 2 is built without these taxa. Color code: Brucellaceae, red; Bartonellaceae, light green; Phyllobacteriaceae, pink; Rhizobiaceae, royal blue; Aurantimonadaceae, orange; Rhodobacteraceae, dark purple; Bradyrhizobiaceae, light blue; Xanthobacteraceae, yellow; Methylobacteriaceae, coral; Methylocystaceae, dark green; Beijerinckiaceae, brown. Naming abbreviations given in S1 Table. (TIF) [file pone.0135655.s002.tif]

Mirrored comparison 1:  
ExoR (left) vs ExoS (right)

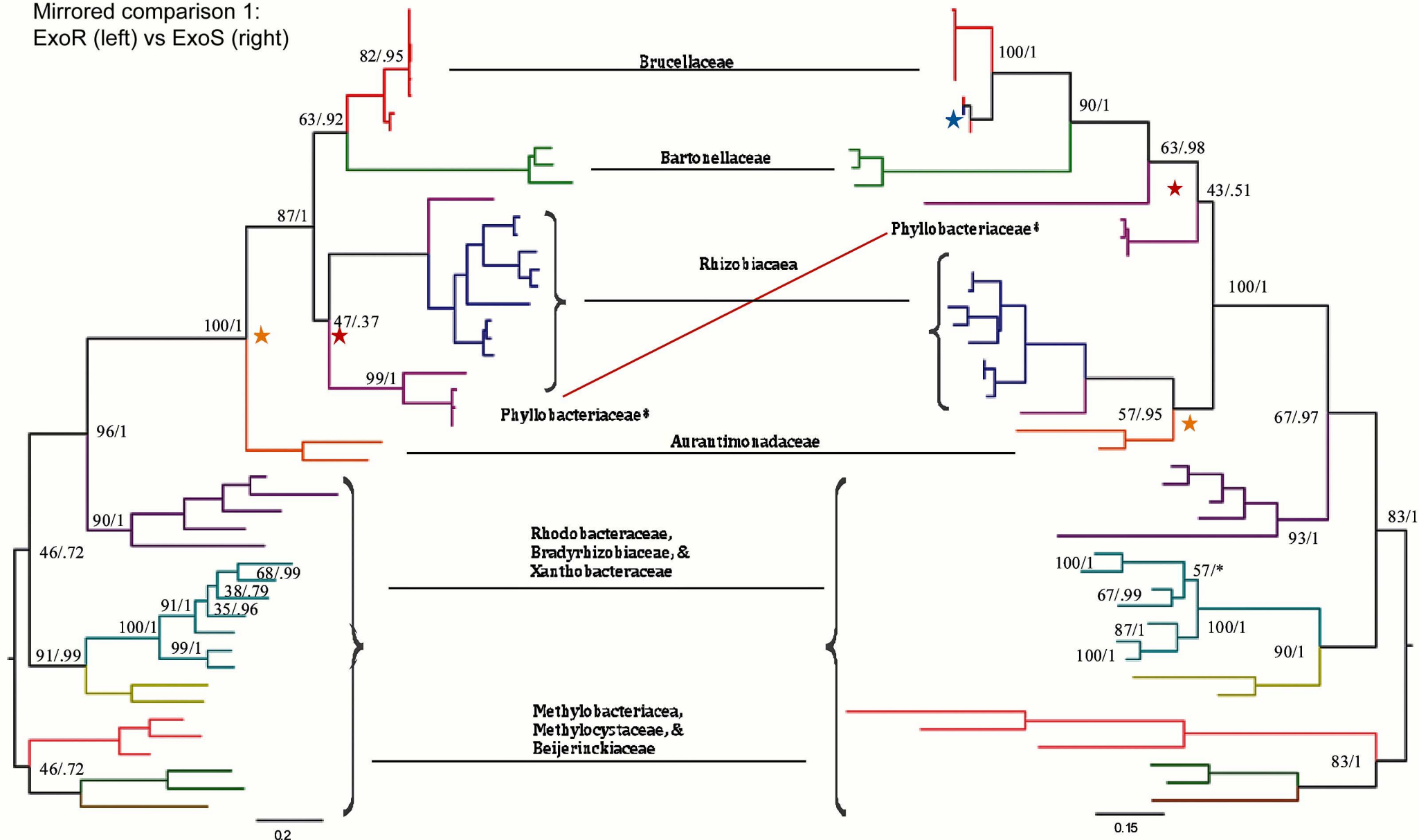

Mirrored comparison 2:  
ExoS (left) vs ChvI (right)

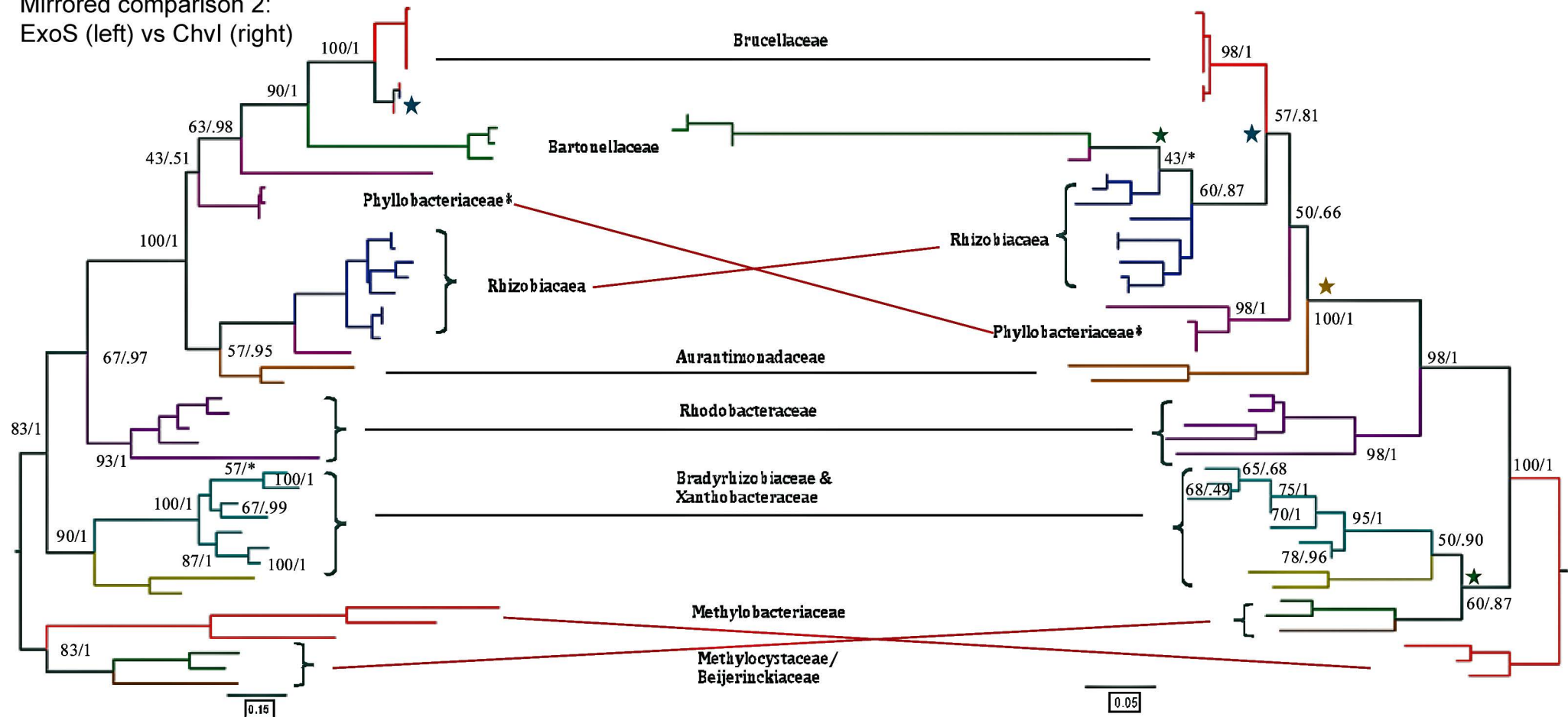

Supplement: S3 Fig — (ExoR orthologs on left ExoS on right, first figure; ExoS orthologs on left and ChvI on right, second figure) Similar branching patterns among the RSI switch proteins suggest a unified phylogenetic history. Bootstrap values, followed by posterior probabilities, are given. Starred nodes are non-congruent. Color codes given in S2 Fig. (PDF) [file pone.0135655.s003.pdf]
