# Supplementary material for: Phylogenetic Co-Occurrence of ExoR, ExoS, and ChvI, Components of the RSI Bacterial Invasion Switch, Suggests a Key Adaptive Mechanism Regulating the Transition between Free-Living and Host-Invading Phases in Rhizobiales
Source: PLoS One. 2015 Aug 26;10(8):e0135655. doi: 10.1371/journal.pone.0135655 (PMC4550343; doi:10.1371/journal.pone.0135655)
Supplement: S2 Table — Results of from reciprocal BLASTs and predictions of signal peptides, domains, and transmembrane helices (TMH). (PDF) [file pone.0135655.s005.pdf]

Column1	Column2	Column3	Column5	Column6	Column7	Column8	Column9	Column10
**Supplemental Table S2. Analysis of putative ExoR homologs**							**TMH predictions**	
**Genome of origin, ExoR-like proteins**	**Abbrev. in trees**	**Order**	**Length (residues)**	**Reciprocal blast test**	**Sequence characterizations**	**Signal peptide &/or localization predictions**	**(only positive predictions indicated)**	**# of Sel1 predictions**
**Ortholog candidates**								
Afipia.sp.1NLS2	Afipia	Rhizobiales	271	Passes		Yes		4
Agrobacterium.radiobacter.K84	AgrbcRad	Rhizobiales	267	Passes		Yes		2
Agrobacterium.sp.H13-3	AgrbcSp	Rhizobiales	264	Passes		Yes		4
Agrobacterium.tumefaciens.str.C58	AgrbcTu	Rhizobiales	264	Passes		Yes		4
Agrobacterium.vitis.S4	AgrbcVi	Rhizobiales	271	Passes		Yes		4
Aurantimonas.manganoxydans.SI85-9A1	AurntmMg	Rhizobiales	278	Passes		Yes		4
Bartonella.grahamii.as4aup	BartnGrm	Rhizobiales	263	Passes		Yes		3
Bartonella.henselae.str.Houston-1	BartnHen	Rhizobiales	264	Passes	Potential non-classical secretion	SP vs TMH: No consensus		5
Bartonella.tribocorum.CIP.105476	BartnTri	Rhizobiales	264	Passes	Potential non-classical secretion	SP vs TMH: No consensus		3
Bradyrhizobiaceae.bacterium.SG-6C	BrdyrhzB	Rhizobiales	277	Passes		Yes		4
Bradyrhizobium.japonicum.USDA.110	BrdyrhzJ	Rhizobiales	297	Passes		Yes		4
Bradyrhizobium.sp.BTAi1	BrdyrhzS	Rhizobiales	240	Passes	Potential non-classical secretion	Yes		4
Brucella.abortus.bv.1.str.9-941 (REF)	BrucA99	Rhizobiales	267	Passes		Yes		4
Brucella.abortus.str.2308A	BrucA23	Rhizobiales	268	Passes		Yes		6
Brucella.ceti.str.Cudo	BrucCeti	Rhizobiales	268	Passes		Yes		5
Brucella.neotomae.5K33	BrucNeo	Rhizobiales	268	Passes		Yes		4
Brucella.sp.BO2	BrucSp	Rhizobiales	268	Passes		Yes		4
Brucella.suis.1330 (REF)	BrucSu	Rhizobiales	267	Passes		Yes		4
Chelativorans.sp.BNC1 (aka Agrobacterium.sp.BNC1)	Cheltiv	Rhizobiales	268	Passes		Yes		4
Fulvimarina.pelagi.HTCC2506	FulviPel	Rhizobiales	262	Passes		Weak SP predictions		4
Hoeflea.phototrophica.DFL-43	HoefPHot	Rhizobiales	265	Passes		Yes		4
Labrenzia.alexandrii.DFL-11	LabrenzI	Rhodobacterales	238	Passes	Potential non-classical secretion	No		4
Mesorhizobium.ciceri.biovar.biserrulae.WSM1271	MsorhzCi	Rhizobiales	279	Passes		Yes		4
Mesorhizobium.loti.MAFF303099	MsorhzLo	Rhizobiales	276	Passes		Yes		4
Mesorhizobium.opportunistum.WSM2075	MsorhzOp	Rhizobiales	283	Passes		Yes		4
Methylobacterium.chloromethanicum.CM4	MthlobCh	Rhizobiales	295	Passes		Yes		4
Methylobacterium.radiotolerans.JCM.2831	MthlobRad	Rhizobiales	300	Passes		Yes		4
Methylobacterium.sp.4-46	MthlobSp	Rhizobiales	269	Passes		Yes		4
Methylocella.silvestris.BL2	Mthlocel	Rhizobiales	280	Passes		Yes		4
Methylocystis.sp.ATCC.49242	Mthlocyst	Rhizobiales	296	Passes		Yes		4
Methylosinus.trichosporium.OB3b	MthlsinT	Rhizobiales	275	Passes		Yes		4
Nitrobacter.hamburgensis.X14	Nitrobac	Rhizobiales	273	Passes		Yes		4
Ochrobactrum.anthropi.ATCC.49188	OchroAn	Rhizobiales	263	Passes		Yes		4
Ochrobactrum.intermedium.LMG.3301	OchroIn	Rhizobiales	262	Passes		Yes		4
Oligotropha.carboxidovorans.OM5	OliboCarb	Rhizobiales	275	Passes		Yes		4
Polymorphum.gilvum.SL003B-26A1	PolymoGil	Rhodobacterales	280	Passes		Yes		2
Pseudovibrio.sp.JE062	PseudovSp	Rhodobacterales	249	Passes	Potential non-classical secretion	No		2
Rhizobium.etli.CFN.42	RhizobEt	Rhizobiales	265	Passes		Yes		4
Rhizobium.leguminosarum.bv.trifolii.WSM1325	RhizobLeg	Rhizobiales	267	Passes		Yes		4
Rhodopseudomonas.palustris.CGA009 (REF)	Rhodopseu	Rhizobiales	273	Passes		Yes		4
Roseibium.sp.TrichSKD4	Roseibium	Rhodobacterales	266	Passes		Boarderline SP predictions		2
Sinorhizobium.fredii.NGR234	SinoFred	Rhizobiales	268	Passes		Yes		4
Sinorhizobium.medicae.WSM419	SinoMed	Rhizobiales	267	Passes		Yes		4
Sinorhizobium.meliloti.1021	SinoMel	Rhizobiales	268	N/A		Yes		4
Stappia.aggregata.IAM12614 (aka Labrenzia.aggregata.IAM12614)	LabrenzA	Rhodobacterales	285	Passes		SP		4
Starkeya.novella.DSM.506	Starkeya	Rhizobiales	279	Passes		SP		2
Xanthobacter.autotrophicus.Py2	Xanthobac	Rhizobiales	310	Passes		Boarderline	Boarderline	4
**Outlying structural or localization predictions**								**Avg = 4**
Azorhizobium.caulinodans.ORS.571		Rhizobiales	351	Passes		*No*	*TMH prediction*	4
Beijerinckia.indica.subsp.indica.ATCC.9039		Rhizobiales	342	Passes		*No*	*TMH prediction*	3
Novosphingobium.aromaticivorans.DSM12444		Sphingomonadales	432	Passes	*SPOR domain (PF05036)*	*No*	*Weak TMH prediction*	2
Parvibaculum.lavamentivorans.DS-1		Rhizobiales	278	Passes		*No*	*TMH prediction*	4
Pelagibacterium.halotolerans.B2		Rhizobiales	382	Passes		*No*	*Weak TMH prediction*	2
**Phylogenetically non-resolvable with current methods**								**Avg = 3**
Ahrensia.sp.R2A130		Rhodobacterales	287	Passes		Yes	*Weak TMH prediction*	4
Hyphomicrobium.denitrificans.ATCC.51888		Rhizobiales	370	Passes		Yes		0
Hyphomicrobium.sp.MC1		Rhizobiales	339	Passes		Yes		3
Magnetospirillum.magnetotacticum.MS-1		Rhodospirillales	260	Passes	Potential non-classical secretion	No		4
Rhodomicrobium.vannielii.ATCC17100		Rhizobiales	391	Passes		Boarderline	*Weak TMH prediction*	4
**Non-orthologous**								**Avg = 4**
Acetobacter.pasteurianus.IFO3283-01 (REF)		Rhodospirillales	590	Fails		No		7
Asticcacaulis.excentricus.CB_48		Caulobacterales	497	Fails	Putative PodJL			7
Asticcacaulis.excentricus.CB_48		Caulobacterales	417	Fails				4
Azospirillum.brasilense.Sp245		Rhodospirillales	567	Fails	Putative β-lactamase	No		13
Azospirillum.lipoferum.4B		Rhodospirillales	450	Fails	Putative PodJL ortholog	No		6
Azospirillum.sp.B510		Rhodospirillales	452	Fails	Putative PodJL ortholog	No		6
Gluconacetobacter hansenii ATCC 23769		Rhodospirillales	606	Fails		No		7
Gluconacetobacter.diazotrophicus.PA1_5		Rhodospirillales	593	Fails				12
Gluconacetobacter.diazotrophicus.PA1_5		Rhodospirillales	593	Fails				11
Gluconacetobacter.europaeus.LMG18494		Rhodospirillales	594	Fails		No SP consensus		8
Gluconacetobacter.xylinus.NBRC3288		Rhodospirillales	595	Fails				11
Gluconobacter.oxydans.621H		Rhodospirillales	590	Fails		No SP consensus		8
Granulibacter.bethesdensis.CGNIH1		Rhodospirillales	652	Fails		No SP consensus		9
Hyphomicrobium.sp.MC1		Rhizobiales	360	Fails				4
Hyphomicrobium.sp.MC1		Rhizobiales	384	Fails				5
Magnetococcus.marinus.MC-1		Magnetococcales	831	Fails		No SP consensus		14
Magnetococcus.marinus.MC-1		Magnetococcales	976	Fails		No SP consensus		12
Magnetospirillum.gryphiswaldense.MSR-1		Rhodospirillales	471	Fails				8
Magnetospirillum.magneticum.AMB-1		Rhodospirillales	393	Fails	Putative PodJL ortholog			6
Maricaulis.maris.MCS10		Rhodobacterales	401	Fails				4
Maricaulis.maris.MCS10		Rhodobacterales	1072	Fails	PG_binding_1 domain (PF01471)	No		3
Octadecabacter.arcticus.238		Rhodobacterales	455	Fails	Potential non-classical secretion	*No*		4
Parvularcula.bermudensis.HTCC2503		Parvularculales	411	Fails				6
Rhodobacter.sphaeroides.2.4.1 (REF)		Rhodobacterales	971	Fails	Peptidase_C14 domain (PF00656)			9
Rhodobacter.sphaeroides.ATCC 17025		Rhodobacterales	400	Fails		No	TMH prediction	5
Rhodomicrobium.vannielii.ATCC17100		Rhizobiales	598	Fails	Possibly not secreted			9
Rhodospirillum.photometricum.DSM122		Rhodospirillales	262	Fails		No		7
Rickettsia bellii OSU 85-389		Rickettsiales	1359	Fails				5

Roseobacter.sp.SK209-2-6		Rhodobacterales	782	Fails	Peptidase_C14 (PF00656) & PG_binding_1 (PF01471) domains			6
Sphingobium.chlorophenolicum.L-1		Sphingomonadales	261	Fails	Putative PodJL ortholog			4
Sphingobium.japonicum.UT265		Sphingomonadales	345	Fails	SPOR domain (PF05036)			2
Sphingobium.sp.SYK-6		Sphingomonadales	357	Fails	SPOR domain (PF05036)			2
Sphingopyxis.alaskensis.RB2256		Sphingomonadales	396	Fails	SPOR domain (PF05036)			2
Sphinogomonas.wittichii.RW1		Sphingomonadales	250	Fails				4
Zymomonas.mobilis.subsp.mobilis.ATCC10988 (REF)		Sphingomonadales	455	Fails				11

Notes: CSD = Cat-scratch disease
