# Supplementary material for: Phylogenetic Co-Occurrence of ExoR, ExoS, and ChvI, Components of the RSI Bacterial Invasion Switch, Suggests a Key Adaptive Mechanism Regulating the Transition between Free-Living and Host-Invading Phases in Rhizobiales
Source: PLoS One. 2015 Aug 26;10(8):e0135655. doi: 10.1371/journal.pone.0135655 (PMC4550343; doi:10.1371/journal.pone.0135655)
Supplement: S3 Table — Every Alphaproteobacterial genome with an identified ExoR ortholog also encodes ExoS and ChvI orthologs. Many of these species are facultatively intracellular and include well-known examples of both pathogens and symbionts. (PDF) [file pone.0135655.s006.pdf]

Lifestyle characterizations of Alphaproteobacteria encoding RSI orthologs

Bacteria	Protein Tree Abbreviation	Order	Family	Pathogen?	Significant Roles	Lifestyle	Habitat
Afipia sp.1NLS2	Afipia	Rhizobiales	Brady-rhizobiaceae		N2 ox./ Bioremediation		
Agrobacterium radiobacter K84	AgrbcRad	Rhizobiales	Rhizobiaceae	Plant: Crown Gall		Facultative pathogen	
Agrobacterium sp. H13-3	AgrbcSp	Rhizobiales	Rhizobiaceae	Plant: Crown Gall		Facultative pathogen	
Agrobacterium tumefaciens str C58	AgrbcTu	Rhizobiales	Rhizobiaceae	Plant: Crown Gall; Human: Opportunist	Biotechnology	Facultative pathogen	
Agrobacterium vitis S4	AgrbcVi	Rhizobiales	Rhizobiaceae	Plant: Crown Gall		Facultative pathogen	
Aurantimonas manganoxydans SI85-9A1	AurntmMg	Rhizobiales	Auranti-monadaceae		Mn(II) oxidation		Marine, oxic/anoxic
Bartonella grahamii as4aup	BartnGrm	Rhizobiales	Bartonellaceae	Mammalian: Intra-erythrocitic		Facultative pathogen	
Bartonella henselae str. Houston-1	BartnHen	Rhizobiales	Bartonellaceae	Mammalian: Intra-erythrocitic		Facultative pathogen	
Bartonella tribocorum CIP 105476	BartnTri	Rhizobiales	Bartonellaceae	Mammalian: Intra-erythrocitic		Facultative pathogen	
Bradyrhizobiaceae bacterium SG-6C	BrdyrhzB	Rhizobiales	Brady-rhizobiaceae				
Bradyrhizobium japonicum USDA 110	BrdyrhzJ	Rhizobiales	Brady-rhizobiaceae		N2 fixation (soy)		
Bradyrhizobium sp. BTAi1	BrdyrhzS	Rhizobiales	Brady-rhizobiaceae		N2 fixation (non-legume)		
Brucella abortus bv 1 str 9-941	BrucA99	Rhizobiales	Brucellaceae	Mammalian: Brucellosis		Facultative pathogen	

Brucella abortus str 2308A	BrucA23	Rhizobiales	Brucellaceae	Mammalian: Brucellosis		Facultative pathogen	
Brucella ceti str Cudo	BrucCeti	Rhizobiales	Brucellaceae	Mammalian: Brucellosis		Facultative pathogen	
Brucella neotomae 5K33	BrucNeo	Rhizobiales	Brucellaceae				
Brucella sp. BO2	BrucSp	Rhizobiales	Brucellaceae	Mammalian: Brucellosis		Facultative pathogen	
Brucella suis 1330	BrucSu	Rhizobiales	Brucellaceae	Mammalian: Brucellosis		Facultative pathogen	Marine
Chelativorans sp. BNC1 (aka *Agrobacterium sp* BNC1)	Cheltiv	Rhizobiales	Phyllo-bacteriaceae		Bioremediation		Soil, sewage
Fulvimarina pelagi HTCC2506	FulviPel	Rhizobiales	Auranti-monadaceae		Mn(II) oxidation		Marine
Hoeflea phototrophica DFL-43	HoefPHot	Rhizobiales	Phyllo-bacteriaceae			Associates w/ dinoflagellates	Marine
Labrenzia alexandrii DFL-11	LabrenzI	Rhodo-bacterales	Rhodo-bacteraceae			Associates w/ dinoflagellates	Marine
Labrenzia aggregata IAM12614	LabrenzA	Rhodo-bacterales	Rhodo-bacteraceae		Denitrification		Marine
Mesorhizobium cicero biovar biserrulae WSM1271	MsorhzCi	Rhizobiales	Phyllo-bacteriaceae		N2 fixation	Facultative symbiont	
Mesorhizobium loti MAFF303099	MsorhzLo	Rhizobiales	Phyllo-bacteriaceae		N2 fixation	Facultative symbiont	
Mesorhizobium opportunistum WSM2075	MsorhzOp	Rhizobiales	Phyllo-bacteriaceae		N2 fixation	Facultative symbiont	
Methylobacterium chloromethanicum CM4	MthlobCh	Rhizobiales	Methylo-bacteriaceae		Bioremediation		Soil, sewage, leaf surfaces

Methylobacterium radiotolerans JCM 2831	MthlobRad	Rhizobiales	Methylo-bacteriaceae	Mammalian: Nosocomial bacteremia	Radiation tolerant	Facultative pathogen	Soil, sewage, leaf surfaces
Methylobacterium sp. 4-46	MthlobSp	Rhizobiales	Methylo-bacteriaceae		Methanotroph		Soil, sewage, leaf surfaces
Methylocella silvestris BL2	Mthlocel	Rhizobiales	Beijerinckiaceae		Methanotroph		Soil, acidophile
Methylocystis sp. ATCC 49242	Mthlocyst	Rhizobiales	Methylo-cystaceae		NH3 ox./ Methanotroph		Aquatic, acidic soil
Methylosinus trichosporium OB3b	MthlsinT	Rhizobiales	Methylo-cystaceae		N2 fix./ Methanotroph		
Nitrobacter hamburgensis X14	Nitrobac	Rhizobiales	Brady-rhizobiaceae		NO2 oxidization		Soil, freshwater
Ochrobactrum anthropic ATCC 49188	OchroAn	Rhizobiales	Brucellaceae	Mammalian: Emergent nosocomial		Facultative pathogen	
Ochrobactrum intermedium LMG 3301	OchroIn	Rhizobiales	Brucellaceae	Mammalian: Emergent nosocomial		Facultative pathogen	
Oligotropha carboxidovorans OM5	OliboCarb	Rhizobiales	Brady-rhizobiaceae		CO2 fixation		Soil, sewage
Polymorphum gilvum SL003B-26A1	PolymoGil	Rhodo-bacterales	Rhodo-bacteraceae		Bioremediation		Halophilic
Pseudovibrio sp. JE062	PseudovSp	Rhodo-bacterales	Rhodo-bacteraceae			Facultative marine symbiont	
Rhizobium etli CFN 42	RhizobEt	Rhizobiales	Rhizobiaceae		N2 fixation	Facultative symbiont	
Rhizobium leguminosarum bv trifolii WSM1325	RhizobLeg	Rhizobiales	Rhizobiaceae		N2 fixation	Facultative symbiont	
Rhodopseudomonas palustris CGA009	Rhodopseu	Rhizobiales	Brady-rhizobiaceae		N2/CO2 fixation		Soil, aquatic

Roseibium sp. TrichSKD4	Roseibium	Rhodo-bacterales	Rhodo-bacteraceae		Carbon fixation	Cyanobact. symbiont	Marine
Sinorhizobium fredii NGR234	SinoFred	Rhizobiales	Rhizobiaceae		N2 fixation	Facultative symbiont	
Sinorhizobium medicae WSM419	SinoMed	Rhizobiales	Rhizobiaceae		N2 fixation	Facultative symbiont	
Sinorhizobium meliloti 1021	SinoMel	Rhizobiales	Rhizobiaceae		N2 fixation	Facultative symbiont	
Starkeya novella DSM 506	Starkeya	Rhizobiales	Xantho-bacteraceae		S oxidation/ Methanotroph		Soil
Xanthobacter autotrophicus Py2	Xanthobac	Rhizobiales	Xantho-bacteraceae		Alkenotrophic		

Candidate ExoR orthologs with divergent structural predictions							
Azorhizobium caulinodans ORS 571	TM helices, lacks localization prediction consensus	Rhizobiales	Xantho-bacteraceae		N2 fixation	Facultative symbiont	Soil
Beijerinckia indica subsp indica ATCC 9039	TM helices, lacks localization prediction consensus	Rhizobiales	Beijerinckiaceae		N2 fix./ Biotechnology		Soil
Novosphingobium aromaticivorans DSM12444	No SP prediction; SPOR domain (PF05036)	Sphingo-monadales	Sphingo-monadaceae	Mammalian: biliary cirrhosis	Xenobiotic degradation	Facultative pathogen	
Parvibaculum lavamentivorans DS-1	TM helices, lacks localization prediction consensus	Rhizobiales	Phyllo-bacteriaceae		Bioremediation		Marine
Pelagibacterium halotolerans B2	TM helices, lacks localization prediction consensus	Rhizobiales	Hypho-microbiaceae			Extreme tolerance to salinity & heavy metals	Marine
