# Supplementary material for: Phylogenetic Co-Occurrence of ExoR, ExoS, and ChvI, Components of the RSI Bacterial Invasion Switch, Suggests a Key Adaptive Mechanism Regulating the Transition between Free-Living and Host-Invading Phases in Rhizobiales
Source: PLoS One. 2015 Aug 26;10(8):e0135655. doi: 10.1371/journal.pone.0135655 (PMC4550343; doi:10.1371/journal.pone.0135655)
Supplement: S1 Table — GenBank accession numbers & BLAST results. (PDF) [file pone.0135655.s004.pdf]

Column1<br>Supplemental Table S1. Final ortholog datasets	Column2	Column3	Column4	Column5	Column6	Column7	Column8	Column9	Column10	Column11
	Abbrev. in trees	Order	Family	ExoR GenBank	ExoR phmmer E	ExoS GenBank	ExoS phmmer E	ChvI GenBank	ChvI phmmer E	Characterisitics
**Genus.species.strain**										
Afipia.sp.1NLS2	Afipia	Rhizobiales	Bradyrhizobiaceae	ZP_07028141.1	3.0E-36	ZP_07025826.1	9.8E-64	ZP_07025825.1	2.7E-119	Haloacetic acid degrader, denitrifier
Agrobacterium.radiobacter.K84	AgrbcRad	Rhizobiales	Rhizobiaceae	YP_002544306.1	3.0E-36	YP_002542762.1	CS 8e-65	YP_002542763.1	NCBI nr 2e-149	Crown gall causative agent
Agrobacterium.sp.H13-3	AgrbcSp	Rhizobiales	Rhizobiaceae	YP_004278964.1	1.0E-37	YP_004277350.1	2.3E-111	YP_004277351.1	1.8E-31	Crown gall causative agent
Agrobacterium.tumefaciens.str.C58	AgrbcTu	Rhizobiales	Rhizobiaceae	NP_354703.1	2.5E-121	NP_353072.1	9.0E-112	NP_353073.2	3.2E-26	Crown gall causative agent
Agrobacterium.vitis.S4	AgrbcVi	Rhizobiales	Rhizobiaceae	YP_002549936.1	3.6E-119	YP_002548010.1	3.6E-110	YP_002548009.1	2.2E-138	Grapevine crown gall causative agent
Aurantimonas.manganoxydans.SI85-9A1	AurntmMg	Rhizobiales	Aurantimonadaceae	ZP_01227286.1	3.8E-65	ZP_01228482.1	4.7E-89	ZP_01228481.1	4.6E-27	Marine Mn(II) oxidizing bacteria, oxic/anoxic envirnoments
Bartonella.grahamii.as4aup	BartnGrm	Rhizobiales	Bartonellaceae	YP_002972246.1	1.5E-58	YP_002971124.1	1.0E-64	YP_002971125.1	7.0E-118	Facultatively intracellular pathogen, rodent reservior, ocular involvement in disease
Bartonella.henselae.str.Houston-1	BartnHen	Rhizobiales	Bartonellaceae	YP_033846.1	1.5E-58	YP_032926.1	1.6E-64	YP_032927.1	4.7E-119	Agent of bacillary angiomatosis, CSD, bacillary peliosis endocarditis
Bartonella.tribocorum.CIP.105476	BartnTri	Rhizobiales	Bartonellaceae	YP_001609870.1	1.1E-52	YP_001608557.1	5.0E-65	YP_001608558.1	1.4E-117	Facultatively intracellular pathogen, rodent reservior
Bradyrhizobiaceae.bacterium.SG-6C	BrdyrhzB	Rhizobiales	Bradyrhizobiaceae	ZP_08629727.1	2.3E-34	ZP_08627521.1	1.3E-66	ZP_08627520.1	5.1E-119	Chemolitoautotrophic bacterium/taxonomy not established
Bradyrhizobium.japonicum.USDA.110	BrdyrhzJ	Rhizobiales	Bradyrhizobiaceae	NP_771402.1	8.93-32	NP_774785.1	2.0E-59	NP_774784.1	1.0E-117	Superior nitrogen fixation on soy
Bradyrhizobium.sp.BTAi1	BrdyrhzS	Rhizobiales	Bradyrhizobiaceae	YP_001240397.1	6.6E-32	YP_001243138.1	1.7E-60	YP_001243137.1	4.6E-117	Nodulates a non-legume (Parasponia) host
Brucella.abortus.bv.1.str.9-941 (REF)	BrucA99	Rhizobiales	Brucellaceae	YP_221610.1	6.0E-77	YP_222727.1	5.7E-77	YP_222726.1	2.5E-129	Cattle Brucellosis, facultative intracellular pathogen
Brucella.abortus.str.2308A	BrucA23	Rhizobiales	Brucellaceae	ZP_04594300.1	1.8E-78	ZP_04595435.1	5.7E-77	YP_415418.1	2.5E-129	Cattle Brucellosis, facultative intracellular pathogen
Brucella.ceti.str.Cudo	BrucCeti	Rhizobiales	Brucellaceae	ZP_03785397.1	9.4E-79	ZP_03784511.1	1.5E-77	ZP_03784510.0	2.5E-129	Brucellosis, isolated from cetaceans, facultative intracellular pathogen
Brucella.neotomae.5K33	BrucNeo	Rhizobiales	Brucellaceae	ZP_05964176.1	4.7E-77	ZP_05963229.1	1.5E-77	ZP_05963228.1	4.3E-129	Wood rat Brucellosis, facultative intracellular pathogen
Brucella.sp.BO2	BrucSp	Rhizobiales	Brucellaceae	ZP_07473132.1	5.0E-77	ZP_07475436.1	1.6E-77	EFM58577.1	2.5E-129	Brucellosis, potentially human, facultative intracellular pathogen
Brucella.suis.1330 (REF)	BrucSu	Rhizobiales	Brucellaceae	YP_05615709.1	3.5E-77	NP_699066.1	1.5E-77	NP_699065.1	2.5E-129	Porcine Brucellosis, facultative intracellular pathogen
Chelativorans.sp.BNC1 (aka Agrobacterium.sp.BNC1)	Cheltiv	Rhizobiales	Phyllobacteriaceae	YP_674247.1	2.9E-74	YP_676106.1	5.7E-59	YP_676105.1	3.4E-128	Degrades synthetic chelators (EDTA), isolated from soil & sewage
Fulvimarina.pelagi.HTCC2506	FulviPel	Rhizobiales	Aurantimonadaceae	ZP_01440116.1	1.0E-54	ZP_01437653.1	2.5E-89	ZP_01437652.1	5.1E-23	Phototrophic marine Mn(II) oxidizer, xanthorhodopsin & photosynthetic genes
Hoeflea.phototrophica.DFL-43	HoefPhot	Rhizobiales	Phyllobacteriaceae	ZP_02166546.1	7.6E-108	ZP_02164588.1	6.3E-101	ZP_02164587.1	5.7E-137	Marine, associated with dinoflagellates, containing bacteriochlorophyll a
Labrenzia.alexandrii.DFL-11	LabrenzI	Rhodobacterales	Rhodobacteraceae	ZP_05117387.1	1.9E-33	ZP_05115329.1	1.9E-81	ZP_05115286.1	1.4E-121	Marine, associated with dinoflagellates, containing bacteriochlorophyll a
Mesorhizobium.ciceri.biovar.biserrulae.WSM1271	MsorhzCi	Rhizobiales	Phyllobacteriaceae	YP_004142654.1	2.7E-70	YP_004139690.1	2.6E-84	YP_004139691.1	3.1E-128	Nodulates drought-tolerant field legume Biserrula
Mesorhizobium.loti.MAFF303099	MsorhzLo	Rhizobiales	Phyllobacteriaceae	NP_102763.1	2.4E-70	NP_105816.1	6.9E-85	NP_105817.1	2.6E-128	Nodulates model legume *Lotus japonicus*
Mesorhizobium.opportunistum.WSM2075	MsorhzOp	Rhizobiales	Phyllobacteriaceae	YP_004612503.1	1.7E-70	YP_004608985.1	2.5E-85	AEH84890.1	1.8E-50	Newly emerged, nodulates Australian field legume, B. pelecinus
Methylobacterium.chloromethanicum.CM4	MthlobCh	Rhizobiales	Methylobacteriaceae	YP_002421006.1	2.1E-30	YP_002420208.1	3.9E-47	YP_002420209.1	5.7E-125	Dechlorination and metabolism of chloromethane
Methylobacterium.radiotolerans.JCM.2831	MthlobRad	Rhizobiales	Methylobacteriaceae	YP_001755590.1	3.4E-31	YP_001755753.1	1.3E-40	YP_001755754.1	3.0E-123	Radiation tolerant, bacteremia causative species
Methylobacterium.sp.4-46	MthlobSp	Rhizobiales	Methylobacteriaceae	YP_001771818.1	6.9E-31	YP_001767233.1	2.5E-52	YP_001767232.1	2.1E-124	Pink-pigmented facultative methylotrophs, isolated from soil and plants
Methylocella.silvestris.BL2	Mthlocel	Rhizobiales	Beijerinckiaceae	YP_002361994.1	5.0E-33	YP_002360596.1	1.8E-59	YP_002360597.1	7.0E-117	Acidophilic aerobic faciltative soil methanotroph
Methylocystis.sp.ATCC.49242	Mthlocyst	Rhizobiales	Methylocystaceae	ZP_08070851.1	6.3E-33	ZP_08071160.1	2.0E-62	ZP_08071161.1	7.7E-120	Methanotroph, oxidizes ammonia, aquatic and acidic soil environments
Methylosinus.trichosporium.OB3b	MthlsinT	Rhizobiales	Methylocystaceae	ZP_06887308.1	3.9E-31	ZP_06887046.1	1.8E-61	ZP_06887047.1	6.9E-120	Obligate methanotroph with soluble monooxygenase, N2 fixation
Nitrobacter.hamburgensis.X14	Nitrobac	Rhizobiales	Bradyrhizobiaceae	YP_577028.1	1.0E-32	YP_575792.1	1.0E-61	YP_575793.1	2.7E-118	Facultative chemolithoautotroph, oxidizes nitrite, soil & freshwater
Ochrobactrum.anthropi.ATCC.49188	OchroAn	Rhizobiales	Brucellaceae	YP_001370899.1	7.7E-80	YP_001369378.1	3.2E-79	YP_001369379.1	9.7E-131	Emerging pathogen, nonsocomial, colonizer of humans and plants
Ochrobactrum.intermedium.LMG.3301	OchroIn	Rhizobiales	Brucellaceae	ZP_04680080.1	4.5E-80	ZP_04681465.1	2.6E-80	ZP_04681464.1	6.2E-130	Opportunistic pathogen closely related to *Brucella* spp.
Oligotropha.carboxidovorans.OM5	OligoCarb	Rhizobiales	Bradyrhizobiaceae	YP_002289255.1	2.0E-35	YP_002290542.1	CS 2e-58	YP_002290541.1	NCBI nr 2e-137	Chemolithoautotrophic, utilizes CO, fixes CO2, soil & wastewater bacterium
Polymorphum.gilvum.SL003B-26A1	PolymoGil	Rhodobacterales	Rhodobacteraceae	YP_004304078.1	3.3E-23	YP_004305928.1	6.9E-78	YP_004305929.1	9.5E-121	Crude oil degradation, halophilic
Pseudovibrio.sp.JE062	PseudovSp	Rhodobacterales	Rhodobacteraceae	ZP_05082905.1	8.4E-38	ZP_05084217.1	5.4E-61	ZP_05084204.1	9.1E-121	Marine sponge symbionts
Rhizobium.etli.CFN.42	RhizobEt	Rhizobiales	Rhizobiaceae	YP_468274.1	8.4E-38	YP_467595.1	2.5E-111	YP_467596.1	1.2E-137	Common bean (*P. vulgaris*) nodulator
Rhizobium.leguminosarum.bv.trifolii.WSM1325	RhizobLeg	Rhizobiales	Rhizobiaceae	YP_002975502.1	9.0E-115	YP_002978074.1	9.2E-111	YP_002978075.1	6.5E-139	Meditteranean, Australian clover microsymbiont
Rhodopseudomonas.palustris.CGA009 (REF)	Rhodopseu	Rhizobiales	Bradyrhizobiaceae	NP_948203.1	1.4E-33	NP_945710.1	5.2E-67	NP_945711.1	4.9E-118	Capable of four modes of metabolism, fixes CO2 and N2, soil & water bacterium
Roseibium.sp.TrichSKD4	Roseibium	Rhodobacterales	Rhodobacteraceae	ZP_07662196.1	3.4E-32	ZP_07658178.1	4.3E-76	ZP_07658177.1	1.1E-118	Isolated from marine cyanobacteria uses DMSP as carbon source
Sinorhizobium.fredii.NGR234	SinoFred	Rhizobiales	Rhizobiaceae	YP_002825893.1	9.2E-146	YP_002827875.1	9.8E-127	YP_002827876.1	4.8E-142	Legume nodulator with very broad host range
Sinorhizobium.medicae.WSM419	SinoMed	Rhizobiales	Rhizobiaceae	YP_001326839.1	3.3E-153	YP_001328910.1	2.0E-131	YP_001328911.1	5.1E-151	Close relative to Sm1021, legume nodulator
Sinorhizobium.meliloti.1021	SinoMel	Rhizobiales	Rhizobiaceae	NP_385624.1	N/A	NP_384149.1	N/A	NP_384150.1	N/A	Close relative to *S. medicae* , legume nodulator
Stappia.aggregata.IAM12614	LabrenzA	Rhodobacterales	Rhodobacteraceae	ZP_01546515.1	1.1E-34	ZP_01547991.1	7.3E-81	ZP_01547990.1	2.1E-121	Marine, no bacteriochlorophyll a, reduces nitrate & nitrite
Starkeya.novella.DSM.506	Starkeya	Rhizobiales	Xanthobacteraceae	YP_003693818.1	2.4E-39	YP_003696261	2.3E-70	YP_003696260.1	2.9E-118	Facultatively chemolithoautotrophic, sulfur-oxidizing, methanotrophic soil bacterium
Xanthobacter.autotrophicus.Py2	Xanthobac	Rhizobiales	Xanthobacteraceae	YP_001419116.1	2.4E-39	YP_001417743.1	6.8E-70	YP_001417742.1	1.1E-117	Facultative alkenotroph, enzymes encoded on megaplasmid
Total										48
Notes: CSD = Cat-scratch disease										
CS = Context Specific Blast										
NCBI = Species not available in phmmer; E value from Blastp										
